# From Unmet Medical Need to Drug Candidate: A Translational Therapeutic Development Roadmap Illustrated by Dual-Payload Antibody–Drug Conjugates

**DOI:** 10.3390/biom16071052

**Published:** 2026-07-18

**Authors:** Takeshi Honda, Gui-Dong Zhu

**Affiliations:** SparX Biopharmaceutical Corp., 700 E Business Center Dr, Mount Prospect, IL 60056, USA; takeshi.honda@sparxbio.com

**Keywords:** antibody–drug conjugate, dual-payload ADC, drug resistance, payload pairing, topoisomerase I inhibitor, target product profile, translational oncology, molecular combination therapy, tumor heterogeneity, developability

## Abstract

Transformative therapeutic innovation should not begin with a molecule—or even a molecular target. It should begin with a clearly defined unmet clinical need. Here, we present a seven-step Translational Therapeutic Development Roadmap that systematically connects an unmet medical need to a developable drug candidate through the disciplined sequence of (i) defining the need, (ii) understanding disease and resistance biology, (iii) building a mechanistic hypothesis, (iv) defining a target product profile (TPP), (v) molecular design and experimental validation, (vi) developability and manufacturability assessment, and (vii) clinical translation. A central conclusion emerging from this review is that resistance biology should be viewed not merely as a cause of therapeutic failure, but as a primary design input for next-generation therapeutic innovation. Our analysis identifies continuous alignment among unmet clinical needs, resistance biology, mechanistic hypothesis, molecular design, developability, and clinical translation as the defining characteristic of successful therapeutic development. We use dual-payload antibody–drug conjugates (ADCs) as a contemporary and highly illustrative case study of this resistance-informed therapeutic development approach. Single-payload ADCs such as trastuzumab deruxtecan and sacituzumab govitecan have transformed treatment across multiple solid tumors, yet most patients ultimately relapse through antigen loss, defective intracellular trafficking, drug efflux, payload-target alterations, and tumor heterogeneity, creating an emerging post-ADC treatment gap. Dual-payload ADCs, which deliver two mechanistically distinct warheads from a single antibody, represent a form of molecular combination therapy designed to increase the barrier to resistance and address multiple escape pathways simultaneously, as well as provide a clinically relevant model for resistance-informed therapeutic design. Using dual-payload ADCs as a worked example, we demonstrate how resistance biology directly informs payload pairing, molecular architecture, conjugation strategy, experimental validation, and developability. Our analysis indicates that successful dual-payload ADC design depends not simply on combining two cytotoxic payloads, but on selecting complementary mechanisms with non-overlapping resistance liabilities while satisfying predefined target product profiles and manufacturability requirements. We further summarize resistance-guided payload pairing strategies, including topoisomerase I plus tubulin inhibitors, topoisomerase I plus DNA-damage-response inhibitors, cytotoxic plus immunomodulatory payloads, and cell-permeable plus non-permeable combinations; the conjugation chemistries that enable defined dual-payload products; the preclinical validation, pharmacological optimization, and developability hurdles that separate promising biology from viable therapeutics; and the rapidly expanding clinical landscape, including the first-in-human program KH815 and emerging bispecific dual-payload constructs. Finally, we demonstrate that the same translational roadmap extends beyond ADCs to radiopharmaceutical conjugates, multispecific antibodies, targeted protein degraders, and cell and gene therapies, indicating that it represents a general framework for therapeutic innovation rather than an ADC-specific strategy. Collectively, this review supports the concept that therapeutic innovation is most successful when unmet clinical needs, resistance biology, molecular design, developability, and clinical translation are considered as an integrated continuum rather than as independent stages of drug discovery. This Translational Therapeutic Development Roadmap provides an organizing framework for guiding the rational development of next-generation targeted therapeutics across diverse therapeutic modalities.

## 1. PART I—A Translational Principles Underlying Resistance-Informed Therapeutic Innovation

### 1.1. Introduction: Why Therapeutic Innovation Begins with Unmet Medical Needs

Over the past two decades, biomedical research has transformed our understanding of cancer through advances in cancer genomics, next-generation sequencing, single-cell and spatial transcriptomics, proteomics, structural biology, CRISPR-based functional genomics, computational chemistry, artificial intelligence (AI), and systems biology. Together, these technologies have revealed the molecular complexity, cellular heterogeneity, and evolutionary dynamics of human cancers while dramatically accelerating target discovery and therapeutic design. In parallel, therapeutic modalities have expanded far beyond conventional cytotoxic chemotherapy to include molecularly targeted therapies, immune checkpoint inhibitors, antibody–drug conjugates (ADCs), bispecific antibodies, radiopharmaceuticals, targeted protein degraders, RNA therapeutics, gene editing, engineered cell therapies, and other precision medicine approaches. Among these emerging modalities, ADCs have rapidly evolved into a major therapeutic platform across multiple solid tumors, driven by continuous advances in antibody engineering, payload chemistry, linker technologies, biomarker-guided patient selection, and clinical translation [1]. Collectively, these scientific and technological advances have greatly expanded the opportunities for therapeutic innovation. However, they have also shifted the primary challenge in drug discovery from generating molecular solutions to selecting the right biological problems to solve.

Despite these remarkable advances, the overall success rate of oncology drug development remains disappointingly low. Many investigational therapeutics fail during clinical development because resistance biology, tumor heterogeneity, pharmacological limitations, developability, manufacturability, and clinical implementation are often considered only after candidate molecules have already been identified. Consequently, technological sophistication alone cannot guarantee clinical success. Rather, successful therapeutic innovation requires a systematic translational process that begins with a clearly defined unmet clinical need and continuously integrates biological understanding with rational therapeutic design throughout development.

The history of cancer therapeutics is often told as a story of molecules—of cytotoxins, kinase inhibitors, monoclonal antibodies, and increasingly sophisticated targeted agents. A more accurate telling places the patient first: nearly every enduring therapeutic advance began not with a chemical structure, but with a precisely defined clinical problem that existing therapies could not adequately solve [2,3]. The therapeutic molecule is the answer; the unmet clinical need is the question.

Although the scientific literature frequently focuses on molecular innovations, the process through which successful therapeutics emerge is often less explicitly discussed. In practice, modern drug development follows a translational progression that begins with the identification of an unmet medical need, proceeds through mechanistic understanding of disease and resistance biology, and ultimately culminates in the design, validation, manufacture, and clinical translation of a therapeutic candidate. Making this process explicit provides not only a conceptual framework for understanding therapeutic innovation but also a practical roadmap for guiding future drug discovery.

We hypothesize that successful therapeutic innovation follows a common translational logic that is largely independent of molecular modality. Whether developing small molecules, biologics, ADCs, radiopharmaceuticals, targeted protein degraders, RNA therapeutics, or engineered cell therapies, successful therapeutic development programs generally begin by identifying an unmet clinical need, defining the biological mechanisms responsible for therapeutic failure, translating those insights into a mechanistic hypothesis, and designing therapeutic candidates that directly address those biological limitations. We further propose that resistance biology should be viewed not merely as a cause of therapeutic failure, but as a primary design input for next-generation therapeutic innovation.

The central concept developed in this review is that successful therapeutic innovation requires continuous alignment among unmet clinical needs, resistance biology, mechanistic hypothesis, molecular design, developability, and clinical translation. We present a seven-step Translational Therapeutic Development Roadmap as an organizing framework for this resistance-informed approach. Rather than focusing on a specific therapeutic modality, the roadmap emphasizes the systematic decision-making process that links biological understanding to therapeutic design and ultimately to clinical benefit.

Accordingly, this review uses dual-payload antibody–drug conjugates (ADCs) as a contemporary and highly illustrative example of resistance-informed therapeutic innovation. Through this case study, we demonstrate how understanding mechanisms of therapeutic failure can guide payload selection, molecular architecture, validation strategy, developability assessment, and clinical translation. The Translational Therapeutic Development Roadmap is presented as an organizing framework to describe this integrated decision-making process. Consequently, dual-payload ADCs provide an ideal contemporary example of how unmet clinical needs can be translated into biological understanding, mechanistic hypothesis, molecular architecture, and therapeutic design.

**Figure 1:**
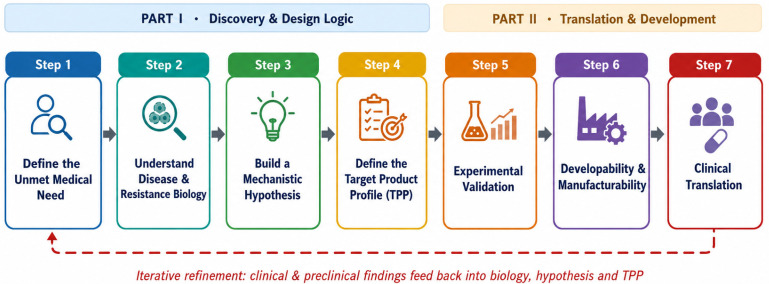
**The Translational Therapeutic Development Roadmap.** The roadmap comprises seven sequential but iterative steps connecting an unmet medical need to a clinical drug candidate. Steps 1–4 (Part I) establish the discovery and design logic; steps 5–7 (Part II) govern translation and development. Dashed feedback arrows indicate that preclinical and clinical findings continually refine the underlying biology, hypothesis, and target product profile.

Viewed through this lens, dual-payload ADCs are more than a new molecular format. They represent an instructive model for resistance-informed therapeutic innovation, illustrating how unmet medical needs, biological understanding, molecular engineering, and translational strategy can be integrated into a coherent therapeutic development pathway. Although illustrated using dual-payload ADCs, the proposed Translational Therapeutic Development Roadmap is intended as a broadly applicable framework that may guide the rational development of radiopharmaceutical conjugates, multispecific biologics, targeted protein degraders, RNA therapeutics, gene-editing strategies, engineered cell therapies, and other emerging precision medicines. Figure 1 summarizes the overall architecture of the proposed roadmap, which serves as the organizational framework for the remainder of this review.

#### 1.1.1. The Evolution of Modern Therapeutics

The evolution of modern cancer therapy can be viewed as a series of responses to unresolved clinical limitations. Cytotoxic chemotherapy established the proof of principle that systemic agents can eradicate disseminated malignancy and, in selected settings, achieve cure. However, its therapeutic index is fundamentally constrained by the shared vulnerabilities of rapidly dividing normal and malignant cells, resulting in dose-limiting toxicities and incomplete tumor selectivity [1,4]. These limitations created a clear unmet need for therapies capable of distinguishing malignant cells from normal tissue.

The molecular characterization of oncogenic drivers transformed this landscape and ushered in the era of targeted therapy. Agents such as imatinib for BCR-ABL-driven leukemia, EGFR inhibitors for mutant non-small-cell lung cancer, and BRAF inhibitors for V600E-mutant melanoma demonstrated that exploiting tumor-specific molecular dependencies could produce dramatic clinical responses while reducing systemic toxicity [5,6,7]. Yet, the success of these agents revealed a new challenge: despite impressive initial responses, most patients eventually developed resistance through secondary mutations, pathway reactivation, or adaptive tumor evolution.

The recognition that cancer progression is influenced not only by tumor-intrinsic pathways but also by host immune regulation led to the next major therapeutic advance. Immune checkpoint inhibitors targeting CTLA-4 and PD-1/PD-L1 demonstrated that releasing endogenous immune restraints could generate durable, and in some cases curative, responses across multiple tumor types [8,9,10]. Nevertheless, a substantial proportion of patients either fail to respond or eventually relapse, highlighting the continued need for more effective and broadly applicable therapeutic approaches.

Antibody–drug conjugates (ADCs) emerged as a fourth therapeutic pillar by combining the selectivity of monoclonal antibodies with the potency of highly cytotoxic payloads [11,12,13,14,15,16,17,18,19]. ADCs address a central limitation of both chemotherapy and targeted therapy by delivering potent cytotoxic agents directly to tumor cells while minimizing systemic exposure. The clinical success of agents such as trastuzumab deruxtecan and sacituzumab govitecan has validated this strategy and expanded the reach of targeted therapy to broader patient populations. However, the same clinical experience has revealed new challenges, including acquired resistance, tumor heterogeneity, and the limited durability of response. These emerging limitations have become the driving force behind the development of next-generation ADCs, including dual-payload ADCs, which seek to address multiple resistance mechanisms simultaneously.

Viewed collectively, this progression illustrates a recurring pattern in therapeutic innovation: each generation of therapy arises not simply because new technologies become available, but because the limitations of existing therapies expose new unmet clinical needs. Understanding this cycle provides the conceptual foundation for the Translational Therapeutic Development Roadmap presented in this review.

#### 1.1.2. Why Successful Therapies Eventually Fail

No matter how elegant the mechanism, durable single-agent control is the exception rather than the rule in advanced malignancies. Across targeted therapies, immunotherapies, radiopharmaceuticals, and ADCs, three interconnected forces repeatedly limit long-term clinical benefit. First, resistance. Tumors acquire or select alterations that diminish therapeutic activity, including target mutation, pathway reactivation, adaptive signaling rewiring, impaired intracellular processing, and drug efflux [20,21,22,23]. Although the specific mechanisms differ between therapeutic modalities, the emergence of resistance is a near-universal feature of cancer treatment.

Second, heterogeneity. Tumors are not homogeneous populations of cells but dynamic ecosystems composed of genetically, epigenetically, and phenotypically distinct subclones [24,25]. A therapy directed against a single target or mechanism may effectively eliminate sensitive populations while leaving behind resistant or less-sensitive subpopulations capable of driving relapse.

Third, evolutionary selection. Every therapeutic intervention acts as a selective pressure. Cells that survive treatment gain a relative fitness advantage and become the foundation for subsequent tumor progression [26,27]. In this sense, therapeutic resistance is not an unexpected event but an anticipated consequence of Darwinian selection operating within a heterogeneous biological system. Importantly, these forces are not unique to oncology. Similar principles underlie antimicrobial resistance, immune escape, and treatment failure across multiple disease areas. Their recurrence suggests that therapeutic innovation should not be viewed as a process of repeatedly discovering new molecules, but rather as a process of systematically identifying and overcoming the biological mechanisms that limit existing therapies.

This perspective forms the conceptual foundation of the Translational Therapeutic Development Roadmap described in this review. If resistance, heterogeneity, and evolutionary adaptation are predictable features of disease biology, then they should be considered at the beginning of therapeutic development rather than after clinical failure has occurred. The most successful next-generation therapies are often those explicitly designed to address the limitations of their predecessors. Dual-payload ADCs have emerged as a contemporary example of this resistance-informed approach to therapeutic innovation and provide an instructive model for the translational principles described in this review.

#### 1.1.3. From Clinical Observation to Therapeutic Innovation

The most generative innovations begin with a clinical observation that exposes a mechanistic gap. The observation that EGFR-mutant lung cancers respond to and then escape first-generation inhibitors via the T790M gatekeeper mutation directly motivated third-generation, mutation-selective inhibitors. In the ADC field, the observation that patients relapsing after one topoisomerase I (TOP1) inhibitor ADC derive limited benefit from a second, mechanistically similar ADC—so-called payload cross-resistance—exposes a gap that mechanistically diversified successors must fill [28,29]. The discipline of converting such observations into design requirements, rather than into incremental line-extensions, is what separates true innovation from iteration.

#### 1.1.4. Dual-Payload ADCs as a Model System for Translational Therapeutic Development

Dual-payload ADCs provide a useful illustrative case because they sit at the intersection of biology, chemistry, and product engineering [30,31]. Their rationale is explicitly resistance-driven: to deliver two mechanistically distinct warheads from a single targeting antibody with the goal of reducing cross-resistance and maintaining activity against heterogeneous tumor subclones [30,31]. Realizing that rationale requires solving conjugation, analytical, and manufacturing problems that epitomize the gap between an attractive hypothesis and a developable medicine. The remainder of this review walks the roadmap in Figure 1 step by step (Part I), then illustrates each step in depth using dual-payload ADCs (Part II), before generalizing the framework to other emerging modalities (Part III).

### 1.2. Step 1: Defining the Unmet Medical Need

#### 1.2.1. Clinical Needs as Drivers of Innovation

The starting point of therapeutic innovation is not a molecular scaffold, a target, or a platform technology, but a clearly defined unmet medical need. Historically, the most successful therapeutic advances have emerged when a clinically important problem was articulated with sufficient precision to guide biological investigation and product design. In this sense, drug discovery begins not with identifying what can be built, but with understanding what remains unsolved.

A well-defined unmet need should be clinically meaningful, biologically grounded, and experimentally testable. It should specify the patient population that remains inadequately served by existing therapies, the therapeutic context in which failure occurs, and the biological mechanisms that are believed to contribute to that failure [32,33]. The most productive need statements are quantitative rather than aspirational: they define who is failing, after what treatment, with what magnitude of residual benefit, and through which dominant mechanisms of resistance or disease progression. Such specificity provides the foundation for all subsequent stages of therapeutic development, including hypothesis generation, target product profile (TPP) definition, molecular design, and clinical translation.

Importantly, unmet medical needs are dynamic rather than static. Every successful therapeutic advance creates new standards of care, which in turn reveal new populations of patients who remain inadequately treated. Consequently, therapeutic innovation can be viewed as a continuous cycle in which clinical success simultaneously exposes the next generation of unresolved problems. Understanding these emerging gaps is therefore the first and most critical step in the Translational Therapeutic Development Roadmap.

#### 1.2.2. Historical Examples of Need-Driven Innovation

The history of oncology provides numerous examples of therapeutic innovation driven by clearly defined clinical needs. The development of EGFR inhibitors addressed the lack of effective targeted therapies for patients with EGFR-mutant non-small-cell lung cancer [6]. BRAF inhibitors emerged in response to the historically poor outcomes associated with BRAF V600E-mutant melanoma [7]. Immune checkpoint inhibitors were developed to address the limited durability of conventional cytotoxic therapies and the need for long-term immune-mediated tumor control [9,10]. Antibody–drug conjugates (ADCs) subsequently emerged as a solution to another longstanding challenge: how to deliver highly potent cytotoxic agents while minimizing systemic toxicity through tumor-selective targeting [11,34].

Viewed collectively, these examples reveal a consistent pattern. Therapeutic breakthroughs rarely arise solely because a new technology becomes available. Rather, they emerge when a specific clinical limitation is identified, the underlying biology is sufficiently understood, and a therapeutic strategy is developed to address that limitation. In each case, the unmet need preceded and ultimately shaped the molecule.

#### 1.2.3. ADCs as a Current Example of Unmet Needs

The clinical success of modern ADCs has itself generated a new unmet medical need. Trastuzumab deruxtecan has transformed the treatment of HER2-positive and HER2-low breast cancers and demonstrated substantial benefit across multiple HER2-expressing malignancies [35,36]. Similarly, sacituzumab govitecan has established a new therapeutic paradigm for triple-negative breast cancer and urothelial carcinoma [37]. These agents have validated ADCs as a major therapeutic modality and expanded the reach of targeted cytotoxic therapy.

Yet, their success has simultaneously exposed important limitations. First, acquired resistance is nearly universal. Although responses can be profound, progression-free survival is typically measured in months, and disease eventually recurs in most patients [28,38]. Second, the durability of response remains limited, reflecting tumor heterogeneity, antigen variability, and payload-specific resistance mechanisms that permit residual tumor populations to survive and re-expand [39]. Third, a clinically significant post-ADC treatment gap has emerged. Patients progressing on one topoisomerase I inhibitor-based ADC frequently exhibit substantial cross-resistance to subsequent ADCs employing related payload classes, leaving few mechanistically distinct therapeutic options [29,40].

This unmet need can therefore be defined with considerable precision: patients who have progressed following treatment with highly active ADCs, particularly topoisomerase I inhibitor-based ADCs, require therapies capable of overcoming resistance mechanisms that compromise current payload classes. Defining this gap—who is failing, after what therapy, and through which biological mechanisms—provides the starting point for the development of next-generation ADCs.

HER2-positive and HER2-low breast cancer exemplify the iterative evolution of targeted therapies. Therapeutic innovation has progressed from trastuzumab and pertuzumab to trastuzumab emtansine and subsequently to trastuzumab deruxtecan, with each generation specifically designed to address limitations of its predecessor [34,41,42]. Nevertheless, even trastuzumab deruxtecan ultimately encounters resistance through mechanisms including HER2 downregulation, altered target engagement, drug-efflux transporters, and adaptive DNA-damage response pathways [38,39]. The limited availability of mechanistically differentiated therapies following progression highlights the need for approaches capable of addressing multiple resistance mechanisms simultaneously. Experimental strategies that alter antigen targeting, diversify payload mechanisms, or combine orthogonal therapeutic pressures represent direct responses to this emerging clinical challenge [40,43].

#### 1.2.4. Characteristics of a High-Value Therapeutic Opportunity

Not all unmet medical needs are equally positioned to generate successful therapeutic innovation. High-value opportunities typically share several characteristics. First, they involve a substantial or rapidly expanding patient population for whom existing therapies provide inadequate benefit. Second, the biological mechanisms underlying therapeutic failure are sufficiently understood to support rational intervention. Third, meaningful clinical endpoints are available to demonstrate therapeutic improvement. Fourth, there exists a plausible path toward a differentiated product profile that offers advantages over current standards of care. Finally, the opportunity must be compatible with a realistic path toward clinical development, manufacturing, and regulatory approval.

### 1.3. Step 2: Understanding Disease and Resistance Biology

Having defined the unmet clinical need, the next step in translational therapeutic development is to determine why existing therapies fail. Clinical observations alone rarely provide sufficient guidance for therapeutic design. Rather, effective innovation requires translating patterns of treatment failure into a mechanistic understanding of the biological processes that limit therapeutic benefit. This process—mapping disease and resistance biology—transforms a clinical problem into a biologically actionable design challenge.

For targeted therapies, including ADCs, resistance is rarely attributable to a single molecular event. Instead, therapeutic failure typically emerges through a network of interacting biological mechanisms operating across multiple levels of tumor organization, ranging from target expression and intracellular trafficking to payload sensitivity and tumor microenvironmental influences. Understanding this network is essential because each resistance mechanism represents both a biological obstacle and a potential therapeutic opportunity.

#### 1.3.1. Mapping Biological Failure Mechanisms

A useful starting point is to view therapeutic activity as a sequence of biological events that must occur successfully for treatment to achieve its intended effect. Every step in this sequence represents a potential point of failure. For ADCs, efficacy depends on a multi-step cascade involving antigen recognition, target binding, internalization, intracellular trafficking, lysosomal processing, payload release, intracellular payload retention, and engagement of the payload’s molecular target. Failure at any stage may reduce therapeutic activity, and resistance mechanisms can emerge at multiple points simultaneously [44,45,46,47].

The objective of biological failure mapping is therefore not simply to catalogue resistance mechanisms but to identify the dominant biological constraints limiting therapeutic efficacy within the patient population defined in Step 1. These constraints ultimately become the design requirements that guide subsequent therapeutic innovation.

#### 1.3.2. Resistance as an Evolutionary Process

Resistance is best understood as an evolutionary process driven by therapeutic selection pressure acting upon pre-existing and treatment-induced biological diversity [23,26]. Tumors are dynamic populations composed of heterogeneous cellular subclones that differ in their genetic, epigenetic, metabolic, and phenotypic characteristics. Therapeutic intervention selectively eliminates sensitive populations while enriching those capable of survival.

Importantly, clinically relevant resistance is frequently polyclonal rather than monoclonal. Multiple resistance mechanisms may emerge simultaneously within different tumor regions or cellular populations. Studies of patients progressing on ADC therapy have documented parallel alterations affecting both antigen recognition and payload sensitivity, demonstrating that multiple escape pathways can be selected concurrently under therapeutic pressure [28].

This evolutionary perspective has important implications for therapeutic design. If resistance arises through multiple independent escape routes, then strategies targeting only a single vulnerability may provide limited durability. Understanding the diversity of potential escape mechanisms therefore becomes a prerequisite for designing therapies capable of producing more sustained clinical benefit.

#### 1.3.3. Biological Resistance Networks

**Figure 2:**
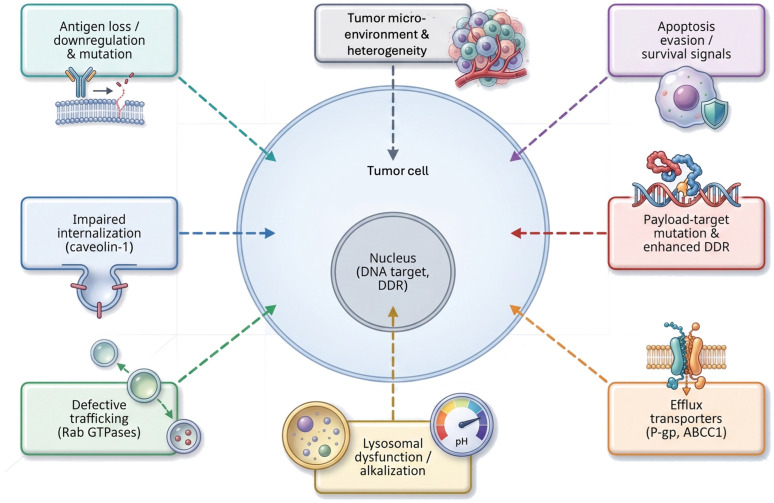
**Biological Mechanisms of ADC Resistance.** Resistance emerges at every stage of the ADC mechanism of action—antigen binding and internalization, endosomal–lysosomal trafficking, payload release, and engagement of the payload’s molecular target—and is reinforced by drug efflux, apoptosis evasion, and the tumor microenvironment. Because escape is typically polyclonal, single-mechanism agents leave multiple routes open; each node also defines an opportunity for rational, multi-mechanistic design.

Resistance to ADCs is not a single lesion but a network (Figure 2). Antigen loss or downregulation, and mutations that impair antibody binding, remove the molecular target required for ADC delivery; loss of HER2 expression and binding is a recurrent driver of trastuzumab deruxtecan resistance [39,48]. Impaired internalization—for example through caveolin-1–dependent endocytic rerouting—reduces productive uptake [49]. Defective intracellular trafficking, including altered Rab-GTPase–controlled endosomal transport and reduced lysosomal proteolytic activity, prevents payload liberation [50,51]. The lysosomal transporter SLC46A3 is required to export certain maytansinoid catabolites, and its loss confers resistance [51,52]. Efflux transporters such as P-glycoprotein (ABCB1) and ABCC1 actively pump released payloads out of the cell, a dominant mechanism for hydrophobic warheads [48,53]. Payload-target resistance—the mutation or upregulation of TOP I, or enhanced DNA-damage-response (DDR) capacity—neutralizes the warhead even when it reaches its target [54,55]. Finally, the tumor microenvironment and spatial heterogeneity shelter antigen-low and poorly perfused subpopulations [24,56].

Several features of this network are decisive for design. First, the nodes are partly independent: a tumor may escape through antigen loss in one region and through enhanced DNA repair in another, so a single counter-measure rarely suffices [47,57]. Second, some nodes are payload-class-specific—efflux substrate status and DNA-repair capacity matter for camptothecin-class warheads, whereas spindle-assembly competence matters for auristatins—which is precisely why combining warheads from different classes is expected to be non-cross-resistant [45,46]. Third, multi-omic profiling of tumors progressing on trastuzumab deruxtecan has confirmed that resistance is convergent yet heterogeneous, enriching alterations that affect HER2 levels, the NRF2/KEAP1 oxidative-stress axis, and ABCC1-mediated efflux—mechanisms a mechanistically diversified conjugate could in principle bypass [38,58]. Mapping which nodes dominate in a given indication is therefore not an academic exercise but the empirical basis for payload selection [59,60].

#### 1.3.4. Translating Biological Insights into Therapeutic Opportunities

The ultimate objective of resistance biology is not simply to explain therapeutic failure, but to identify opportunities for therapeutic innovation. Each resistance mechanism suggests potential design solutions. Efflux-mediated resistance may be addressed through payload diversification or transporter-insensitive payloads. Payload-target resistance may be mitigated through orthogonal mechanisms of action. Antigen heterogeneity may be addressed through bystander-capable payloads, multispecific targeting strategies, or complementary delivery mechanisms. Microenvironmental resistance may motivate incorporation of immune-modulating components [30,61].

Viewed in this way, the resistance network functions as a design specification for the next generation of therapeutics. Every biological limitation identified in Step 2 provides information that can be incorporated into the mechanistic hypothesis developed in Step 3. The emergence of dual-payload ADCs exemplifies this principle: rather than treating resistance as an unavoidable consequence of therapeutic success, dual-payload strategies seek to anticipate and address multiple resistance pathways simultaneously through rational molecular design.

### 1.4. Step 3: Building a Mechanistic Hypothesis

#### 1.4.1. Hypothesis-Driven Drug Discovery

A mechanistic hypothesis is a testable proposition of the form: in this population, failing through this mechanism, an agent with these properties is expected to restore durable control. It is the hinge between biology and molecular design, and it must be specific enough to be wrong [33]. For the post-ADC population, the hypothesis crystallizes as: simultaneously delivering two warheads with orthogonal mechanisms and non-overlapping resistance liabilities may suppress the polyclonal escape that contributes to treatment failure with single-payload ADCs.

#### 1.4.2. Historical Successes of Mechanism-Based Innovation

Several landmark therapeutic advances illustrate how mechanistic understanding can guide drug development. The development of PARP inhibitors in BRCA-deficient tumors was based on the principle of synthetic lethality, whereby simultaneous disruption of complementary DNA repair pathways selectively compromises tumor survival. Similarly, successive generations of EGFR inhibitors were developed in response to specific resistance mutations that emerged during treatment with earlier agents.

ADCs provide another illustrative example. The design of trastuzumab deruxtecan was informed by recognition of tumor heterogeneity and the limitations of earlier HER2-directed therapies. The deruxtecan payload was selected not only for potency but also for membrane permeability and bystander activity, properties that enable killing of neighboring antigen-low cells and potentially broaden activity across heterogeneous tumors [61,62,63,64]. These examples highlight a recurring theme in therapeutic development: molecular properties are often selected to address specific biological constraints rather than simply maximize pharmacological activity.

#### 1.4.3. Combination Therapy as a Response to Resistance

Combination therapy represents one of the most successful historical strategies for overcoming resistance and biological heterogeneity. Across oncology, combinations of agents with distinct mechanisms of action and non-overlapping resistance profiles have repeatedly demonstrated improved durability relative to monotherapy [1,22].

The rationale is straightforward. If resistance to individual therapies occurs independently, simultaneous resistance to multiple orthogonal mechanisms becomes substantially less likely. Combination chemotherapy, targeted therapy combinations, and immunotherapy combinations all exploit this principle. However, conventional combinations are often constrained by cumulative toxicity arising from systemic exposure to multiple active agents, limiting both dosing intensity and duration of treatment.

These limitations have motivated efforts to retain the biological advantages of combination therapy while reducing systemic exposure.

#### 1.4.4. Molecular Combination Therapy

Dual-payload ADCs extend the principles of combination therapy to the molecular level. By incorporating two mechanistically distinct payloads into a single targeted construct, both payloads are delivered to the same cell population through a common antibody-mediated delivery pathway.

This design offers several theoretical advantages. The two payloads are administered in a defined ratio, share pharmacokinetic behavior, and are preferentially delivered to antigen-expressing tissues. Most importantly, cells capable of escaping one mechanism may remain exposed to an alternative therapeutic pressure. In this sense, dual-payload ADCs may be viewed as a form of molecular combination therapy, integrating multiple mechanisms of action within a single targeted agent [30,65,66,67].

#### 1.4.5. Emergence of the Dual-Payload ADC Hypothesis

**Figure 3:**
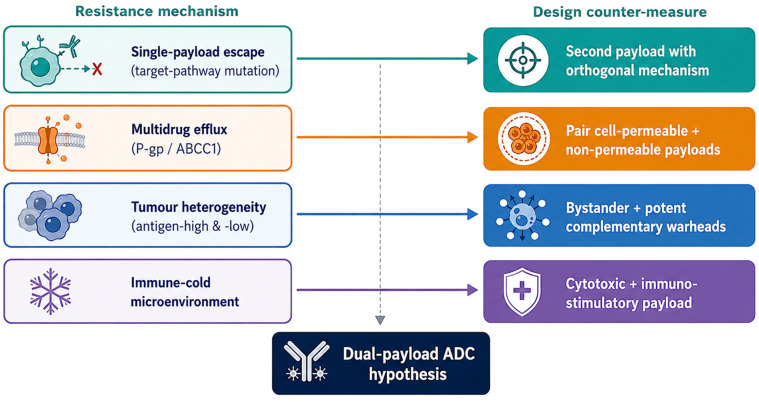
**From resistance mechanisms to therapeutic hypothesis.** Each dominant resistance mechanism on the left maps to a rational design counter-measure on the right; collectively, these converge on the dual-payload ADC hypothesis, in which two warheads with non-overlapping resistance liabilities are co-delivered from a single antibody.

Recent advances in understanding ADC resistance have provided a biological rationale for dual-payload designs. As illustrated conceptually in Figure 3, specific resistance mechanisms may be addressed by corresponding therapeutic design strategies. Payload-target resistance suggests the incorporation of a second payload with an orthogonal mechanism of action. Efflux-mediated resistance motivates pairing payloads with distinct transporter liabilities. Tumor heterogeneity supports combinations of complementary and bystander-capable payloads, while immune-excluded tumor microenvironments provide a rationale for integrating immunomodulatory mechanisms.

Viewed collectively, these observations support the hypothesis that simultaneously addressing multiple resistance pathways may improve the durability of targeted therapy. The emergence of dual-payload ADCs can therefore be viewed not simply as an advance in conjugation technology, but as a biologically informed response to resistance mechanisms identified through clinical and translational research [31,68,69].

Importantly, this framework is intended to illustrate the biological rationale for dual-payload ADC design rather than to imply that all proposed strategies have been clinically validated or will necessarily improve clinical outcomes. The therapeutic performance of any individual dual-payload ADC remains dependent on multiple biological, pharmacological, and developability factors.

### 1.5. Step 4: Defining the Target Product Profile (TPP)

#### 1.5.1. What Is a Target Product Profile?

A target product profile is a structured, prospective statement of the desired characteristics of the final product—indication and line of therapy, efficacy and safety thresholds, dosing, route, and the quality attributes required to support them [32,70]. For biologics and conjugates, a quality TPP (QTPP) additionally fixes critical quality attributes such as the drug-to-antibody ratio (DAR), homogeneity, and stability, which downstream chemistry and manufacturing must deliver [71,72].

#### 1.5.2. Translating Biology into Product Requirements

The TPP is where resistance biology becomes engineering specification. If the need is post-TOP1-inhibitor disease, the TPP must require demonstrable activity in models resistant to that payload class; if efflux is dominant, the TPP must require at least one payload that is not an efflux-pump substrate; if heterogeneity is dominant, the TPP must require bystander activity or dual antigen coverage [30,51,61]. Writing these as pass/fail criteria prevents attractive but non-differentiating molecules from advancing.

#### 1.5.3. Example TPP for Next-Generation ADCs

Table 1 sketches an illustrative TPP for a next-generation dual-payload ADC. Its salient requirements are post-Enhertu activity (objective responses in TOP1-inhibitor-refractory disease), broad resistance coverage (efficacy across antigen-low and efflux-high models), a safety profile compatible with repeat dosing (manageable hematologic and interstitial-lung-disease risk), a convenient dosing schedule, and manufacturability at defined DARs and payload ratios [33,44,71].

In addition to efficacy objectives, the TPP should prospectively define quantitative safety targets, including acceptable exposure margins, anticipated dose-limiting toxicities, and payload-specific toxicity risks. For dual-payload ADCs, the therapeutic objective is not simply to maximize potency but to achieve a predefined balance between efficacy and tolerability.

#### 1.5.4. TPP as the Bridge Between Biology and Molecular Design

The TPP is the contract between discovery and development: it commits the program to deliver specific, testable properties, and it disciplines molecular design by rejecting choices that cannot meet them. With the TPP in hand, the program crosses from Part I (why and what) into Part II (how)—the molecular design, engineering, validation, and translation of dual-payload ADCs [33,71].

## 2. PART II—Dual-Payload ADCs as a Worked Example

### 2.1. Translating Hypothesis into Molecular Design

Having established the clinical need, mapped the underlying resistance biology, and developed a mechanistic hypothesis, the next step is to translate those insights into a therapeutic design. Dual-payload ADCs provide a particularly instructive example because each element of their architecture—including target, payloads, linker, conjugation strategy, and the payload ratio—can be rationally selected in response to specific biological constraints identified in the preceding stages of the roadmap.

#### 2.1.1. Why Two Payloads?

The rationale for dual-payload ADCs follows directly from the polyclonal and multifactorial nature of therapeutic resistance. Tumors rarely fail through a single dominant mechanism; instead, multiple resistant subpopulations frequently coexist within the same lesion or emerge in parallel under treatment pressure [22,28]. Consequently, a therapeutic strategy directed against only one vulnerability may eliminate sensitive populations while leaving alternative escape routes intact.

Theoretical and experimental studies suggest that simultaneous delivery of two payloads with orthogonal mechanisms of action may increase the barrier to resistance by reducing the likelihood that individual tumor cells can evade both therapeutic pressures simultaneously [30,31]. In addition to addressing resistance, payload diversification may broaden activity across heterogeneous tumor populations, including cells occupying different cell-cycle states, expressing different levels of target antigen, or possessing distinct sensitivities to individual payload classes. In this context, dual-payload ADCs can be viewed as an attempt to address biological diversity within tumors through mechanistic diversity within the therapeutic itself.

#### 2.1.2. Why Not Simply Improve a Single Payload?

An important question is whether the same objectives could be achieved through continued optimization of single-payload ADCs. Advances in payload potency, linker stability, bystander activity, and antibody engineering have undoubtedly improved the efficacy of contemporary ADCs and will continue to contribute to future progress.

However, incremental improvements within a single payload class do not necessarily address the fundamental problem of mechanistic cross-resistance. A more potent topoisomerase I inhibitor remains dependent on topoisomerase I engagement and may remain susceptible to resistance mechanisms involving enhanced DNA-damage repair, altered target biology, or transporter-mediated efflux of related catabolites [53,55]. Similarly, increasing payload potency may improve initial tumor killing while leaving the underlying resistance landscape unchanged.

The potential value of dual-payload ADCs therefore derives less from increased potency than from increased mechanistic diversity. Whereas optimization within a payload class typically represents a quantitative improvement, dual-payload approaches seek to introduce a qualitative change in therapeutic behavior by simultaneously targeting multiple resistance pathways.

The greatest advantage of dual-payload delivery is therefore expected in biological contexts where resistance is heterogeneous or multifactorial rather than dominated by a single mechanism. Examples include tumors containing mixed populations with differential payload sensitivity, tumors that acquire multiple resistance pathways under treatment pressure, and post-ADC settings in which cross-resistance limits the activity of a single payload class. In contrast, when resistance is driven predominantly by mechanisms common to all ADCs—such as antigen loss, impaired internalization, or defective intracellular processing—further optimization of targeting or delivery may provide greater benefit than payload diversification. Thus, the value of dual-payload ADCs is likely to depend on matching the molecular design to the dominant biological constraints of the target disease.

#### 2.1.3. Advantages and Limitations of Dual-Payload Strategies

Several potential advantages have driven interest in dual-payload ADCs. By combining payloads with distinct mechanisms of action, dual-payload constructs may suppress or delay resistance, broaden activity across heterogeneous tumors, and integrate complementary pharmacological properties within a single therapeutic. Examples include pairing a potent but non-bystander payload with a membrane-permeable bystander-capable payload, or combining direct cytotoxic activity with immune-modulating mechanisms.

At the same time, these potential benefits are accompanied by significant challenges. The addition of a second payload increases molecular complexity, complicates analytical characterization, and imposes greater demands on conjugation chemistry and manufacturing control [31,73]. The simultaneous delivery of two active mechanisms may also introduce novel or compounded toxicity profiles that are difficult to predict from the individual components alone. Furthermore, unlike conventional combination therapy, the relative ratio of the two payloads is fixed by molecular design and cannot be independently adjusted during clinical treatment.

A critical unresolved question is whether dual-payload ADCs provide benefits beyond those achievable through co-administration of individual agents. In many cases, additive activity may be sufficient to improve resistance coverage and therapeutic breadth. However, the principal rationale for increased molecular complexity is the possibility that co-delivery of complementary payloads may provide therapeutic advantages beyond those achievable through conventional combination therapy under certain biological and pharmacological conditions-and whether such advantages arise from synergy, improved spatial and temporal co-delivery, or other mechanisms remains to be determined experimentally and clinically [74].

An important and often underappreciated limitation of dual-payload ADCs is that the payload ratio is fixed by molecular design. Unlike conventional combination therapy, where the dose of each agent can be independently adjusted during clinical development or treatment, dual-payload ADCs require a single predefined molecular composition to simultaneously optimize efficacy, safety, and therapeutic index. This constraint places greater importance on rational payload pairing, linker design, and payload-ratio optimization during the discovery stage. Consequently, the payload ratio should be selected based on integrated pharmacological, toxicological, and developability considerations rather than on potency alone.

Importantly, dual-payload ADCs are unlikely to overcome all mechanisms of ADC resistance. Their principal advantage lies in addressing resistance associated with payload mechanism, efflux transporters, and intratumoral heterogeneity. In contrast, resistance mechanisms that impair antibody binding, antigen expression, internalization, intracellular trafficking, lysosomal processing, or payload release are expected to compromise both payloads simultaneously. Consequently, dual-payload ADCs should be viewed as a strategy for expanding resistance coverage rather than as a universal solution to ADC resistance.

#### 2.1.4. Dual-Payload ADCs as Molecular Combination Therapy

**Figure 4:**
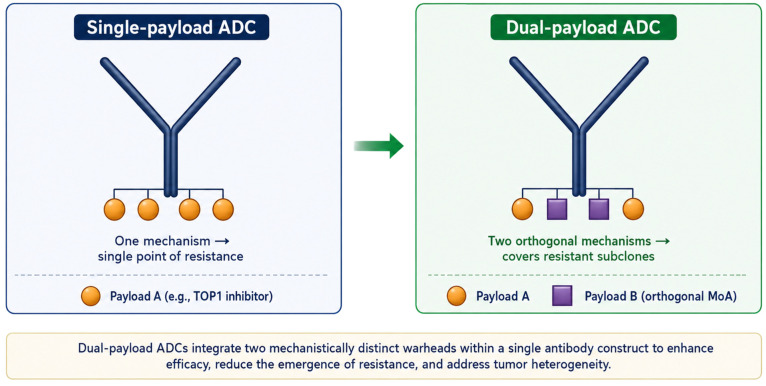
**Single-payload versus dual-payload therapeutic design.** A single-payload ADC delivers one mechanism and therefore presents a single point of resistance. A dual-payload ADC co-delivers two warheads with orthogonal mechanisms of action from the same antibody, behaving as a fixed-ratio, tumor-directed molecular combination therapy that covers resistant subclones. This schematic illustrates conceptual design principles and a hypothesis-generating comparison rather than a clinically validated outcome; any clinical benefit will depend on target expression, internalization, linker release, payload exposure, tolerability, manufacturability, and patient selection.

Conceptually, dual-payload ADCs represent a molecular implementation of combination therapy (Figure 4). Rather than administering multiple agents separately, two mechanistically distinct payloads are incorporated into a single targeted construct and delivered through a shared antibody-mediated delivery pathway.

This architecture offers several potential advantages over conventional combination approaches. Both payloads are designed to be delivered to the same target cell population, maintaining a predefined payload ratio during systemic delivery. Antibody-mediated targeting restricts payload exposure primarily to antigen-expressing tissues, potentially reducing the systemic toxicity that often limits free-drug combinations [30,66]. Most importantly, cells capable of escaping one mechanism may remain susceptible to a second orthogonal mechanism, increasing the likelihood of effective tumor control.

Viewed through this framework, dual-payload ADCs are not simply more complex ADCs. Rather, they represent an emerging class of molecular combination therapies in which multiple therapeutic mechanisms are intentionally integrated within a single targeted agent. Their development reflects a broader trend in oncology toward therapeutic strategies designed not only to achieve tumor killing, but also to anticipate and suppress the biological pathways through which tumors adapt and survive.

Potential advantages over co-administration are expected to be greatest when coordinated delivery to the same target cell, synchronized intracellular exposure, or maintenance of a defined molecular payload ratio is important for therapeutic activity. Conversely, when independent dose optimization of each agent is required, conventional combination therapy may remain the more appropriate strategy. Whether these theoretical advantages translate into superior clinical outcomes remains to be established.

### 2.2. Rational Payload Pairing Strategies

#### 2.2.1. Principles of Resistance-Guided Payload Pairing

The central challenge in dual-payload ADC design is selecting payload combinations that address the biological resistance mechanisms identified in Step 2 while satisfying the target product profile established in Step 4. Consequently, payload selection should not be viewed as a chemistry exercise alone, but as a process of translating resistance biology into molecular design.

The governing principle is orthogonality [21,31]. An effective payload pair should ideally differ not only in mechanism of action but also in resistance liabilities, intracellular processing requirements, transporter susceptibility, and spatial mode of action. In practical terms, the objective is to minimize the likelihood that a single biological adaptation can simultaneously compromise both payloads.

Several criteria emerge from this principle. First, the two payloads should engage distinct molecular targets or biological processes. Second, their dominant resistance mechanisms should be non-overlapping, particularly with respect to drug-efflux transporters and payload-target adaptation. Third, complementary physicochemical properties, such as membrane permeability and bystander activity, may broaden activity across heterogeneous tumor populations. Finally, where possible, the pair should provide additive or synergistic biological activity without introducing unacceptable toxicity.

**Figure 5:**
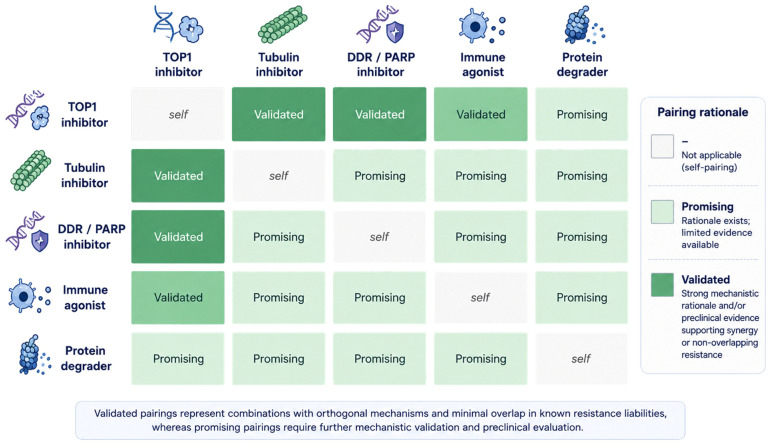
Resistance-guided payload pairing matrix. Pairings of major payload classes are rated by the strength of mechanistic rationale and supporting evidence. The most validated combinations (e.g., topoisomerase I plus tubulin inhibitors, topoisomerase I plus DDR inhibitors) couple warheads with orthogonal targets and non-overlapping resistance mechanisms.

Viewed through the lens of resistance biology, payload pairing becomes a design exercise in coverage. Each payload addresses a subset of resistance mechanisms, and the combined therapeutic objective is to minimize unaddressed escape routes. The following sections review the major pairing strategies currently being explored and the biological rationale supporting each approach [74,75]. Figure 5 summarizes the pairing logic as a matrix of payload classes.

#### 2.2.2. Topoisomerase I + Tubulin Inhibitors

Among currently disclosed dual-payload ADCs, the combination of a topoisomerase I inhibitor and a tubulin inhibitor is the most extensively studied and clinically advanced pairing strategy. Combining a TOP1 inhibitor (e.g., exatecan/DXd) with an anti-tubulin auristatin (e.g., MMAE/MMAF) is the most advanced pairing. The two attack distinct processes—replication-associated DNA damage versus mitotic spindle function—and differ in efflux behavior, so the combination has shown activity in multidrug-resistant and antigen-low models [65,76]. Trastuzumab-based dual conjugates co-delivering MMAE and exatecan have shown high plasma stability, controlled dual release, low aggregation, favorable pharmacokinetics, and enhanced antitumor activity in HER2-low and efflux-overexpressing tumors, designed to address mechanisms associated with resistance to TOP1-inhibitor ADCs such as trastuzumab deruxtecan [31,76].

The pairing also exploits complementary cell-cycle activity. TOP1 inhibitors act predominantly during S-phase by trapping cleavage complexes at replication forks, whereas auristatins arrest cells in mitosis; together they are expected to cover a broader fraction of the cycling population and reduce the pool of cells in a refractory state at any moment [20,77]. Camptothecin-derived warheads have re-emerged as the dominant ADC payload class owing to their combination of moderate potency and bystander activity, which is particularly well suited to heterogeneous solid tumors. However, susceptibility to efflux transporters and DNA damage response (DDR)-mediated resistance remains a recognized limitation. Pairing a topoisomerase I inhibitor with an orthogonal anti-tubulin payload provides a biologically rational strategy that may help to mitigate these resistance mechanisms by reducing reliance on any single cytotoxic pathway [75,78].

Major challenges of development and formulation include cumulative cytotoxicity, overlapping myelosuppression, tubulin-related neuropathy, increased hydrophobicity, aggregation risk, altered pharmacokinetics, and the need to control the ratio and release kinetics of both payloads.

#### 2.2.3. Topoisomerase I + DDR Inhibitors

Whereas TOP1+tubulin pairings primarily seek resistance coverage through mechanistic diversification, TOP1+DDR combinations aim to create biological synergy by simultaneously inducing DNA damage and impairing its repair. Because PARP1 and ATR are essential for repairing TOP1-inhibitor-induced DNA damage, co-delivering a DDR inhibitor with a TOP1 inhibitor can create a synthetic-lethal interaction that may convert otherwise repairable lesions into lethal ones [55,62]. A TOP1 anti-B7-H4 ADC was synergistic with a PARP1-selective inhibitor, exploiting tumor-specific accumulation to localize the synergy, and ratiometric architectures such as SPARC that pre-package TOP1 and DDR inhibitors at defined ratios outperform single-TOP1 ADCs and free-drug combinations [74,75]. Clinical scheduling studies of tumor-targeted TOP1 delivery with optimized PARP inhibition further support the biological rationale for this combination and its therapeutic-window management [79].

The main concern of development and formulation is therapeutic window. DDR inhibitors can sensitize normal proliferating tissues as well as tumor cells. Efficient tumor-selective delivery, stable circulation, controlled intracellular release, and careful dose optimization are essential.

#### 2.2.4. Cytotoxic + Immunomodulatory Payloads

Pairing a cytotoxin with an immune agonist seeks to promote more durable immune-mediated control. Cytotoxic payloads can induce immunogenic cell death, while a co-delivered STING or TLR7/8 agonist activates myeloid cells and remodels an immune-cold microenvironment [80,81]. Immunostimulatory ADCs (iADCs) combining exatecan with a STING agonist, and dual-payload constructs targeting CD276/B7-H3 that elicit both cytotoxicity and immune activation in triple-negative breast cancer, exemplify the approach; a silenced Fc can mitigate Fc-mediated uptake and off-target immune toxicity [68,69]. Co-delivery is designed to concentrates immune activation within the tumor, potentially decoupling efficacy from systemic immune-related toxicity [68]. These designs extend the concept of dual-payload ADCs beyond tumor-cell intrinsic killing and toward the modulation of the broader tumor ecosystem.

Key risks of development include systemic immune activation, cytokine release, liver toxicity, off-tumor uptake by immune cells, and difficulty controlling local versus systemic immune stimulation. Formulation must minimize premature release and preserve immune payload activity after intracellular processing.

#### 2.2.5. Cell-Permeable + Non-Cell-Permeable Payloads

Pairing a membrane-permeable (bystander-competent) payload with a non-permeable, highly potent payload is a rational strategy for addressing intratumoral heterogeneity: the permeable warhead may diffuse into and eliminate antigen-negative neighbors, while the non-permeable warhead delivers high cytotoxic potency to antigen-positive cells while limiting systemic exposure [56,61]. Conjugate homogeneity strongly influences the bystander contribution, underscoring the link between design intent and manufacturing control [67].

An important limitation of this strategy is that bystander-mediated payload diffusion may predominantly expose neighboring antigen-low or antigen-negative cells to only the membrane-permeable payload, while the non-permeable payload remains confined to antigen-positive target cells. Consequently, local single-payload selective pressure may persist in portions of the tumor microenvironment. Whether the overall reduction in tumor burden outweighs this potential evolutionary trade-off remains an important question requiring validation in spatially heterogeneous preclinical models.

#### 2.2.6. Emerging Payload Classes

Beyond classical cytotoxins, three payload classes are entering dual-payload designs. Targeted protein degraders (PROTAC/molecular-glue warheads) delivered as degrader–antibody conjugates can eliminate otherwise undruggable proteins with catalytic, sub-stoichiometric action [82,83,84]. Radiopharmaceutical payloads exploit crossfire radiation to kill antigen-low neighbors independent of internalization [85]. Immune agonists, discussed above, convert local killing into systemic immunity [80]. Each broadens the pairing matrix and the range of resistance mechanisms a conjugate can address.

Targeted inhibitors and degraders are often less intrinsically potent than classical cytotoxic payloads and may re-quire higher intracellular exposure. The released catabolite must retain permeability, target engagement, and degradation activity after lysosomal processing and cytosol escape. Larger molecular size, higher lipophilicity, complex linker design, reduced manufacturability, and biomarker-based patient selection are major challenges.

#### 2.2.7. Is There an Optimal Payload Pair?

Current evidence suggests that no universally optimal payload pair exists. Rather, the optimal combination is context-dependent and determined by the dominant resistance mechanisms, biological characteristics of the target population, and the therapeutic window achievable for a given target and payload combination. There is unlikely to be a single optimal pair; optimality is contextual, defined by the dominant resistance mechanism in the target population and by the achievable therapeutic window [21,31]. The right question is not which pair is best in the abstract but which pair best satisfies the TPP for a specified unmet need—returning the design to the roadmap’s logic. The concept of resistance-guided payload pairing therefore shifts the central question from “Which payload pair is best?” to “Which payload pair best addresses the biological constraints defining the unmet clinical need?”

Importantly, dual-payload ADCs are not expected to overcome all mechanisms of resistance. Their principal advantage lies in addressing resistance at the level of pay-load pharmacology, heterogeneous payload sensitivity, or selected forms of tumor het-erogeneity. Resistance mechanisms occurring upstream of payload action—including complete antigen loss, markedly impaired internalization, defective intracellular trafficking, inefficient lysosomal processing, or insufficient payload release—are unlikely to be overcome solely through payload diversification and may require alternative targeting strategies or therapeutic modalities.

Table 2 summarizes the principal resistance-guided payload pairing strategies dis-cussed above, together with their biological rationale, the resistance mechanisms each is intended to address, their potential advantages, and the major development and formulation challenges that accompany them.

### 2.3. Engineering Dual-Payload ADCs

The biological rationale for dual-payload ADCs is straightforward; their practical implementation is considerably more complex. Unlike conventional single-payload ADCs, dual-payload constructs must control not only total drug loading but also the identity, spatial placement, and relative ratio of two distinct payload species. Consequently, advances in site-specific conjugation, linker engineering, and orthogonal chemistry have been critical enablers of the field. An additional, often underappreciated, challenge is release-kinetic orthogonality. Effective dual-payload ADCs require not only mechanistically distinct payloads but also coordinated intracellular release profiles that preserve the intended pharmacological interaction. Differences in linker cleavage rates, intracellular trafficking pathways, payload retention, and catabolite transport can result in divergent intracellular exposure despite fixed conjugation ratios. Consequently, the biological outcome of a dual-payload ADC may depend as much on the temporal alignment of payload delivery as on the mechanisms of action of the payloads themselves.

Future dual-payload ADC design will therefore likely require optimization of both mechanistic orthogonality and temporal intracellular pharmacology. Such optimization is expected to maximize the likelihood that co-delivered payloads achieve their intend-ed pharmacological interaction in vivo. Importantly, a defined conjugation ratio does not necessarily translate into a defined intracellular exposure ratio. Although both pay-loads share the pharmacokinetic profile of the intact ADC before internalization, their intracellular disposition may diverge because of differences in lysosomal processing, cytosolic escape, intracellular retention, metabolism, and susceptibility to drug-efflux transporters. Accordingly, optimization of dual-payload ADCs should extend beyond the molecular conjugation ratio to include the time-dependent intracellular pharmacology of the released payloads. The design objective is therefore not to preserve a fixed molecular ratio after release, but to achieve the intended temporal pharmacological interaction between the two payloads within the target cell.

In this regard, the emergence of dual-payload ADCs reflects not only a deeper understanding of tumor biology and resistance mechanisms but also substantial advances in ADC engineering technologies. Together, these advances are enabling increasingly sophisticated molecular designs while simultaneously introducing new analytical, manufacturing, and pharmacological challenges that must be addressed during therapeutic development.

#### 2.3.1. Site-Specific Conjugation Technologies

**Figure 6:**
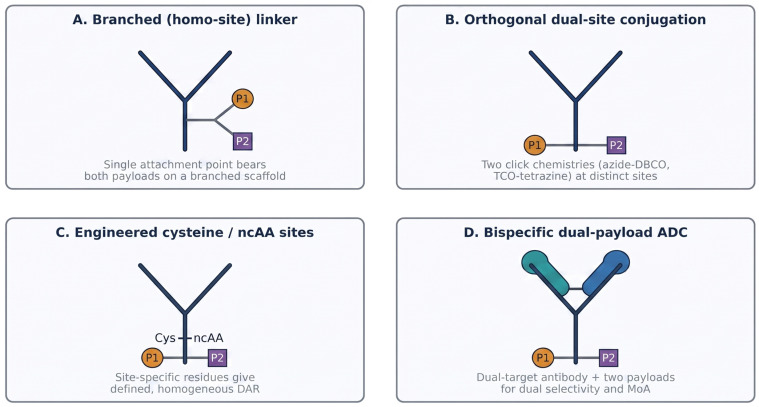
**Engineering strategies for dual-payload ADCs.** (**A**) Branched linkers place both payloads at a single site, decoupling DAR from attachment number. (**B**) Orthogonal dual-site conjugation uses two compatible click chemistries at distinct sites. (**C**) Engineered cysteines or non-canonical amino acids give defined, homogeneous DAR. (**D**) Bispecific dual-payload ADCs combine dual antigen targeting with two mechanistically distinct warheads. Color code: Brown circles indicate Payload 1 (P1), purple squares indicate Payload 2 (P2), green and blue Fab arms represent different antigen-binding domains in the bispecific antibody, and black lines represent the antibody/linker scaffold.

The development of dual-payload ADCs has closely paralleled the evolution of site-specific conjugation technologies. Realizing a defined dual-payload product requires precise control over both the location and number of attached payloads. Engineered cysteine approaches (e.g., THIOMAB, Figure 6C), non-canonical amino acid incorporation (e.g., pAcF and pAMF), and enzymatic conjugation methods have enabled the generation of homogeneous ADCs with defined drug-to-antibody ratios (DARs) and improved therapeutic indices relative to conventional stochastic conjugation [71,86,87,88].

The progression from stochastic to site-specific conjugation is particularly instructive. Traditional conjugation to native lysines or interchain cysteines generates heterogeneous product mixtures spanning multiple DAR species and conjugation sites, often resulting in broader pharmacokinetic distributions and reduced therapeutic windows [89,90]. Site-specific approaches significantly reduce this heterogeneity and have demonstrated improved efficacy and tolerability in head-to-head comparisons with conventional conjugates [71,86]. For dual-payload ADCs, such control is not merely advantageous but essential, as the relative ratio of the two payloads becomes a critical determinant of biological activity, synergy, and reproducibility [31,67].

#### 2.3.2. Branched Linker Architectures

Branched, or multivalent, linker architectures represent one of the most direct approaches for constructing dual-payload ADCs (Figure 6A). These linkers enable multiple payload molecules—or multiple payload classes—to be attached through a single conjugation site, effectively decoupling payload loading from the number of available attachment points [91,92].

The clinical relevance of this strategy is exemplified by KH815, the first dual-payload ADC to enter human clinical testing. KH815 employs a branched linker to co-deliver a topoisomerase I inhibitor and an RNA polymerase II inhibitor on a TROP2-targeting antibody at a combined DAR of approximately 7.5 [93,94]. Such designs illustrate how branched architectures can achieve high payload loading while maintaining defined stoichiometry. However, increasing payload density also increases the risk of aggregation, altered pharmacokinetics, and reduced stability, making linker length, hydrophilicity, and branch geometry important optimization parameters [91].

#### 2.3.3. Orthogonal Conjugation Strategies

An alternative approach involves placing different payloads at distinct sites through orthogonal conjugation chemistries (Figure 6B). In this strategy, two mutually compatible reactions are used sequentially or independently to install separate payloads without cross-reactivity. Examples include combinations of strain-promoted azide–alkyne cycloaddition (SPAAC) and tetrazine–trans-cyclooctene ligation, as well as orthogonal cysteine-protection schemes [65,95].

Orthogonal conjugation can provide greater flexibility than branched-linker approaches because each payload can be independently controlled with respect to loading, release chemistry, and attachment site. The MediLink TMALIN platform exemplifies this concept by conjugating a tubulin-inhibitor payload-linker to specific lysines while simultaneously attaching a topoisomerase I inhibitor payload-linker to engineered cysteine residues, generating a defined dual-payload construct with independently controlled DAR values for each warhead [31,76].

Importantly, the optimal payload ratio cannot be assumed a priori. Rather, it should be regarded as a design variable defined by the target product profile and refined iteratively through mechanistic studies, PK/PD analyses, efficacy, safety, and developability assessments. Because intracellular release kinetics and payload disposition differ between warheads, the optimal conjugation ratio may not necessarily correspond to the optimal intracellular exposure ratio.

#### 2.3.4. Multi-Site and Multi-Payload Platforms

Advances in protein engineering have further expanded the design space available for dual- and multi-payload ADCs. Cell-free expression systems, engineered scaffolds, and site-specific amino acid incorporation technologies allow payloads to be positioned at multiple predefined locations with high precision.

For example, a folate receptor-α-targeting immunostimulatory ADC carrying two immune agonists on the light chains and four cytotoxic payloads on the heavy chains achieved greater than 90% conjugation efficiency while maintaining programmable payload ratios [68,88]. Similarly, ratiometric architectures such as SPARC enable predefined combinations of mechanistically distinct payloads to be delivered in fixed proportions [74]. These studies illustrate that payload stoichiometry can increasingly be treated as a controllable design parameter rather than a manufacturing consequence.

#### 2.3.5. Emerging Bispecific Dual-Payload ADCs

The convergence of dual targeting and dual payload delivery represents an emerging direction in ADC engineering (Figure 6D). These constructs seek to address resistance at both the targeting and payload levels simultaneously by combining multiple antigen-recognition mechanisms with multiple cytotoxic mechanisms.

A representative example is a bispecific EGFR × cMET antibody conjugated to MMAF and SN-38 through a trifunctional linker architecture, which demonstrated superior antitumor activity compared with corresponding single-payload bispecific ADCs and was specifically designed to reduce susceptibility to single resistance mechanisms [96]. Similarly, Alphamab’s JSKN021 combines EGFR/HER3 bispecific targeting with dual-payload delivery of a novel topoisomerase I inhibitor (T01) and MMAE, representing one of the most advanced clinical-stage examples of this strategy [97,98].

Collectively, these developments illustrate a broader trend within ADC engineering: increasing therapeutic complexity is being used not simply to maximize potency, but to address multiple biological vulnerabilities simultaneously. Whether such increasingly sophisticated architectures ultimately translate into superior clinical outcomes remains an important question for the field, but their rapid emergence highlights the growing convergence of resistance biology, molecular engineering, and translational design.

### 2.4. Step 5: Experimental Validation

A mechanistic hypothesis has value only if it generates experimentally verifiable predictions. The purpose of Step 5 is therefore not simply to demonstrate antitumor activity, but to determine whether the proposed therapeutic design successfully addresses the biological limitations that motivated its development. For dual-payload ADCs, validation extends beyond conventional measures of potency and includes explicit evaluation of resistance coverage, tumor heterogeneity, payload interaction, pharmacokinetics, and biomarker associations. Each layer of validation addresses a distinct question in the translational development process, collectively building the evidence required to support clinical translation.

#### 2.4.1. In Vitro Validation

Validation begins by determining whether the biological assumptions underlying the dual-payload design are supported experimentally: potency against antigen-positive lines, evidence of contribution from each payload, and bystander killing of antigen-negative cells in co-culture [56,61]. Demonstrating synergy rather than mere additivity at tolerated payload ratios is the key early readout that justifies the dual design [74,75].

#### 2.4.2. Resistance Models

The defining test is activity in models engineered or selected for the resistance mechanisms identified in Step 2—efflux-pump-overexpressing lines, antigen-low variants, and lines made resistant to the partner single-payload ADC [48,53]. KH815 retained activity in models resistant to sacituzumab govitecan, and the MediLink dual conjugate was active in ABC-transporter-overexpressing and drug-resistant tumors, supporting the hypothesis that the design may overcome the targeted mechanism [76,93].

#### 2.4.3. Heterogeneous Tumor Models

Because heterogeneity motivates the design, validation must include mixed antigen-high/antigen-low populations and spatially heterogeneous xenografts, where bystander-competent and dual-mechanism conjugates are expected to outperform single-payload comparators [61,67].

#### 2.4.4. Patient-Derived Models

Patient-derived xenografts and organoids preserve the molecular and architectural heterogeneity of human tumors and predict clinical response better than cell-line models, making them valuable for de-risking dual-payload candidates before clinical entry [99,100,101].

#### 2.4.5. PK/PD Considerations

Mechanistic, multiscale PK/PD modelling links the disposition of intact conjugate, released payloads, and DAR evolution to efficacy and toxicity, and is essential for dual-payload agents whose two warheads have different release kinetics and systemic exposures [102,103]. Controlled, differential release of the two payloads is both a design goal and a validation endpoint [76]. Unlike single-payload ADCs, dual-payload constructs must maintain not only adequate exposure but also the intended relative exposure of each payload throughout circulation and tumor delivery.

Accordingly, optimization of dual-payload ADCs should consider not only the initial conjugation ratio but also the time-dependent intracellular exposure ratio of released payloads. Experimental quantification of intracellular payload concentrations together with mechanistic PK/PD modelling may therefore become an important component of future dual-payload ADC development.

#### 2.4.6. Biomarker Development

**Figure 7:**
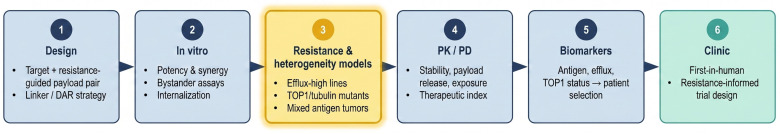
**Preclinical validation workflow.** Candidate dual-payload ADCs progress through layered models—in vitro potency and bystander assays, engineered resistance models, heterogeneous and mixed-population models, patient-derived xenografts and organoids, PK/PD and payload-release studies, and parallel biomarker development—with go/no-go gates that feed back into molecular redesign.

Biomarkers that identify patients most likely to benefit—antigen expression, payload-target status, DDR competence, or efflux-transporter levels—should be developed in parallel with the molecule so that clinical trials can enrich for responders and interpret resistance prospectively [52,104]. Figure 7 summarizes the layered validation workflow.

### 2.5. Step 6: Developability and Manufacturability

The transition from a promising molecule to a clinically viable therapeutic depends not only on biological activity but also on developability. Many mechanistically compelling candidates fail during development because they cannot be reproducibly manufactured, adequately characterized, or formulated into stable pharmaceutical products. For dual-payload ADCs, these challenges are amplified by the need to control two payload species simultaneously while maintaining product consistency, stability, and scalability. Consequently, developability should be viewed not as a downstream consideration, but as an integral component of molecular design.

Developability should therefore include prospective evaluation of the therapeutic window through integrated PK/PD modelling, quantitative toxicology, and exposure–response analyses, allowing unacceptable payload combinations to be eliminated before clinical development.

#### 2.5.1. Product Heterogeneity

Product heterogeneity has long been one of the defining challenges of ADC development. Conventional stochastic conjugation generates distributions of DAR species and conjugation sites, resulting in molecular populations that differ in potency, stability, clearance, and toxicity [72,89]. Dual-payload ADCs introduce an additional layer of complexity because both the distribution of each payload and their relative stoichiometry must be controlled simultaneously [31].

Consequently, product heterogeneity extends beyond total DAR to encompass the composition, distribution, and relative ratio of individual payload combinations. The ability to define and reproducibly control these attributes is fundamental to consistent biological performance, manufacturing robustness, and regulatory acceptance. Unlike conventional ADCs, dual-payload constructs require simultaneous control of the total DAR, individual payload loading, the payload ratio, conjugation-site occupancy, and linker compatibility, all of which directly influence pharmacokinetics, intracellular payload release, efficacy, safety, and batch-to-batch reproducibility.

The successful translation of dual-payload ADCs therefore depends not only on a sound biological rationale but also on robust conjugation chemistry and pharmaceutical engineering. Site-specific conjugation technologies—including engineered cysteine residues, noncanonical amino acid incorporation, enzymatic conjugation, and orthogonal conjugation chemistries—have emerged as key enabling platforms because they provide precise control over payload identity and stoichiometry while minimizing product heterogeneity. The selection of branched-linker or orthogonal conjugation strategies inevitably involves trade-offs among molecular flexibility, analytical tractability, manufacturing robustness, and process scalability.

The increased structural complexity of dual-payload ADCs also places greater demands on analytical characterization. In addition to conventional quality attributes such as antibody identity, purity, and total DAR, comprehensive characterization must establish individual payload distribution, the payload ratio, conjugation-site occupancy, linker integrity, free payload content, aggregation, and product stability throughout manufacturing and storage. No single analytical technique can adequately define these attributes. Instead, orthogonal analytical approaches—including chromatography (HIC, RP-HPLC, and SEC), electrophoresis (CE-SDS), mass spectrometry, peptide mapping, intact-mass analysis, and functional bioassays—are generally required to establish product identity, consistency, stability, and the critical quality attributes (CQAs) expected for regulatory approval.

From a chemistry, manufacturing, and controls (CMC) perspective, the increased molecular complexity of dual-payload ADCs introduces additional challenges in process development, release specifications, stability testing, and regulatory control strategies. Accordingly, manufacturability should not be viewed as a downstream optimization exercise, but as an integral design objective that influences candidate selection from the earliest stages of therapeutic development.

Ultimately, the success of dual-payload ADCs will depend not only on demonstrating biological superiority over conventional ADCs but also on whether their additional molecular complexity can be reproducibly manufactured, comprehensively characterized, and consistently controlled throughout pharmaceutical development. Within the Translational Therapeutic Development Roadmap proposed in this review, developability and manufacturability are therefore integral design constraints—not downstream activities—that should shape molecular architecture, candidate selection, and translational decision-making from the outset.

#### 2.5.2. DAR and Payload Ratio Control

Among the critical quality attributes of dual-payload ADCs, the total drug-to-antibody ratio (DAR) and the payload ratio are the principal molecular design variables governing therapeutic performance. DAR influences potency, pharmacokinetics, aggregation propensity, and tolerability, while the relative ratio of the two payloads directly affects the intended biological interaction between the mechanisms being delivered [51,89]. Consequently, optimization of the DAR and the payload ratio represents not merely a manufacturing objective but a central element of therapeutic design.

Site-specific conjugation technologies and branched-linker architectures have emerged largely to address this challenge. By controlling the location and number of payload attachment sites, these approaches enable reproducible DAR and payload-ratio specifications. The engineered-scaffold immunostimulatory ADC placing immunostimulants and cytotoxins on separate antibody chains provides an example of how payload stoichiometry can be intentionally programmed into molecular design [68,71,91]. In this sense, the payload ratio becomes a deliberate pharmacological design parameter rather than a manufacturing consequence.

Because the DAR and the payload ratio directly influence both efficacy and safety, clinical translation requires robust chemistry, manufacturing, and controls (CMC) strategies that maintain reproducible site occupancy, conjugation homogeneity, total DAR, individual payload ratios, linker stability, and predictable intracellular payload release. Unlike conventional ADCs, where total DAR is often the principal conjugation metric, dual-payload ADCs require simultaneous control of two pharmacologically active species throughout development and manufacturing.

Verification of these attributes relies on orthogonal analytical methods—including hydrophobic interaction chromatography, LC–MS-based intact and subunit analysis, peptide mapping, and payload-specific quantitative assays—to independently quantify each conjugated species and confirm payload-ratio consistency. Stability assessment must further consider not only antibody integrity but also the potential differential release kinetics, intracellular disposition, and pharmacological exposure of the individual payloads under physiological and storage conditions.

Ultimately, the optimal payload ratio should not be regarded as a fixed chemical property but as a biologically defined design objective established by the Target Product Profile (TPP). Within the Translational Therapeutic Development Roadmap proposed in this review, the DAR and the payload ratio therefore represent translational design variables that integrate resistance biology, pharmacology, manufacturability, and clinical objectives into a single molecular architecture.

#### 2.5.3. Analytical Challenges

The analytical characterization of dual-payload ADCs is substantially more demanding than that of conventional ADCs because two independent pharmacologically active payloads must be simultaneously identified, quantified, and controlled throughout development and manufacturing. In addition to confirming antibody identity, purity, and the total drug-to-antibody ratio (DAR), analytical methods must establish payload identity, the payload ratio, site occupancy, conjugation homogeneity, linker integrity, free payload content, aggregation, and product stability throughout development and manufacturing [72].

No single analytical platform is sufficient to resolve this level of molecular complexity. Instead, comprehensive characterization relies on an orthogonal analytical toolkit combining hydrophobic interaction chromatography (HIC), reversed-phase chromatography (RP-HPLC), size-exclusion chromatography (SEC), capillary electrophoresis (CE-SDS), native and denaturing mass spectrometry, peptide mapping, intact-mass analysis, and payload-specific bioassays [105,106]. Collectively, these complementary methods establish product identity, critical quality attributes (CQAs), batch-to-batch consistency, and manufacturing robustness while demonstrating that each manufacturing lot conforms to predefined specifications for the DAR, the payload ratio, conjugation homogeneity, and product stability.

Importantly, analytical characterization should not be viewed solely as a regulatory requirement. Within the Translational Therapeutic Development Roadmap proposed in this review, it provides the experimental evidence that the intended molecular architecture has been reproducibly achieved, thereby linking molecular design, pharmaceutical quality, manufacturing control, and ultimately clinical translation.

#### 2.5.4. Stability and Formulation

A successful ADC must remain stable both during storage and throughout systemic circulation. Premature payload release can narrow the therapeutic window by increasing off-target exposure, whereas physicochemical instability during storage may compromise product quality, manufacturability, and shelf life.

Dual-payload ADCs introduce additional formulation challenges because the two payloads often possess distinct physicochemical properties, including differences in hydrophobicity, reactivity, and release kinetics. These characteristics can increase aggregation propensity and create differential degradation pathways that complicate formulation development. Accordingly, linker selection, payload placement, and formulation optimization are closely intertwined. The trastuzumab MMAE/exatecan dual-payload conjugate provides a representative example in which stability, controlled payload release, and low aggregation were explicitly engineered alongside biological activity [31,76].

#### 2.5.5. Scalability

The practical value of a molecular design ultimately depends on whether it can be manufactured reproducibly at commercial scale. Conjugation strategies that perform well in laboratory settings may encounter significant challenges during process development, technology transfer, and large-scale manufacturing.

Multi-step orthogonal conjugation schemes often provide exceptional molecular control but may introduce additional process complexity, yield loss, and manufacturing risk. In contrast, simpler branched-linker approaches may offer advantages in robustness and scalability despite providing less flexibility in payload placement. Consequently, scalability considerations should be incorporated into candidate selection rather than addressed only after a lead molecule has been identified [90,105,106,107].

#### 2.5.6. Regulatory Considerations

From a regulatory perspective, dual-payload ADCs must satisfy the same fundamental expectations applied to other biopharmaceutical products: identity, purity, potency, safety, and process consistency must be demonstrated through a scientifically justified control strategy [108,109].

The principal regulatory challenge is not the presence of two payloads per se, but the increased structural complexity they introduce, which requires additional control over two active payload species, their relative stoichiometry, and their combined safety profile. Consequently, regulatory assessment requires a clear rationale linking product specifications to the intended Quality Target Product Profile (QTPP), supported by analytical, pharmacological, and toxicological evidence [109,110]. Robust control strategies for the DAR, the payload ratio, and product-related variants will become increasingly important as dual-payload ADCs advance through clinical development.

These regulatory expectations reinforce a broader principle of the Translational Therapeutic Development Roadmap: pharmaceutical quality is not created during regulatory review, but is designed into the molecule throughout development.

#### 2.5.7. Why Good Biology Is Not Enough

A recurring lesson throughout pharmaceutical development is that biological promise alone does not guarantee clinical success. Many therapeutically attractive molecules fail because they cannot be manufactured reproducibly, characterized adequately, or formulated into stable products suitable for human use.

For this reason, developability should be considered alongside biology from the earliest stages of candidate design. Practical factors such as conjugation complexity, manufacturing yield, lot-to-lot consistency, aggregation propensity, analytical tractability, and formulation stability influence the probability of successful translation as profoundly as efficacy itself [90,107].

The discipline imposed by this perspective is to evaluate developability during candidate selection rather than after lead identification. A molecule that exhibits marginally greater potency but requires a fragile multi-step conjugation process may ultimately be less valuable than a slightly less potent candidate built upon a robust, scalable platform. Recognizing and balancing these trade-offs early is a defining characteristic of successful translational development programs and exemplifies the broader principle that, in pharmaceutical development, the process is often inseparable from the product.

### 2.6. Step 7: Clinical Translation

The final stage of the Translational Therapeutic Development Roadmap is clinical translation. Ultimately, the value of any therapeutic hypothesis depends on its ability to improve patient outcomes. For dual-payload ADCs, clinical development represents the first opportunity to determine whether mechanistic diversification translates into more durable tumor control than existing single-payload approaches. This stage therefore serves not only as a test of the molecule, but also as a test of the underlying resistance-informed design philosophy.

#### 2.6.1. Current Clinical Landscape

The rapid emergence of dual-payload ADCs can be viewed as reflecting both scientific progress and evolving clinical needs. More than one hundred ADCs are currently in clinical development worldwide, and dual-payload constructs have moved from conceptual and preclinical studies into early-stage clinical evaluation, with more than a dozen programs disclosed through recent AACR presentations, company reports, and regulatory filings [111,112,113].

This momentum reflects an emerging therapeutic opportunity. As single-payload ADCs continue to move into earlier treatment lines and broader indications, an increasing number of patients are progressing following exposure to highly active ADC therapies. Consequently, therapeutic differentiation increasingly depends on addressing the resistance mechanisms that current ADCs leave behind. Dual-payload designs represent one of the most direct attempts to meet this emerging need [13,33].

At the same time, advances in computational modeling, machine learning, and AI-assisted drug design are beginning to support payload selection, linker optimization, and developability assessment, potentially accelerating the discovery and optimization of increasingly complex ADC architectures [114]. The field is also evolving toward a broader view of therapeutic combinations, in which dual-payload ADCs represent one point along a continuum that includes rational sequencing, combination regimens, and multi-mechanistic therapeutic platforms [44,115,116].

#### 2.6.2. Clinical Programs by Development Stage

The current clinical landscape illustrates the rapid evolution of the field (Table 3). KH815 (Chengdu Kanghong), a TROP2-directed ADC co-delivering a topoisomerase I inhibitor and an RNA polymerase II inhibitor, became the first dual-payload ADC to enter human clinical testing and currently serves as an important early clinical test of the dual- payload ADC concept for the modality [93,94]. Additional programs, including IBI3020 (Innovent), JSKN021 (Alphamab), and several dual-payload and immunostimulatory ADCs from Sutro and collaborators, have progressed into clinical or IND-enabling development [68,93,94,117,118].

Collectively, these programs indicate that dual-payload ADCs are no longer purely experimental concepts. Instead, they now represent a clinically testable therapeutic class spanning multiple targets, payload combinations, and biological rationales.

Importantly, the programs summarized here represent different levels of evidence, ranging from preclinical proof-of-concept to early clinical evaluation. Accordingly, the table is intended to illustrate the diversity of emerging development strategies rather than to imply equivalent levels of clinical validation.

Evidence tiers for Table 3: the listed programs are supported by differing levels of evidence, which should not be interpreted as equivalent. These comprise (i) peer-reviewed clinical data, (ii) registered clinical trials without mature peer-reviewed out-comes, (iii) conference abstracts, (iv) company disclosures and investor communications, and (v) preclinical proof-of-concept. Each program’s development stage should there-fore be interpreted in the context of both its development stage and the maturity of the supporting evidence. * This dual-payload status not confirmed by public sources.

#### 2.6.3. Patient Selection Strategies

Because dual-payload ADCs are intended to address specific resistance contexts, successful clinical development will likely depend on appropriate patient selection. The populations most likely to benefit are those defined during Step 1 of the roadmap: patients progressing following highly active ADC therapies, tumors exhibiting antigen heterogeneity, or tumors harboring resistance mechanisms predicted to compromise existing payload classes [29,40].

Consequently, patient selection should be viewed as an extension of molecular design rather than a separate clinical consideration. A well-designed molecule evaluated in the wrong patient population may fail to demonstrate its intended advantage, whereas appropriate enrichment strategies may reveal meaningful differentiation from existing therapies.

#### 2.6.4. Biomarker-Guided Development

Biomarkers may provide the critical link between biological hypothesis and clinical implementation. Candidate biomarkers include antigen expression, target heterogeneity, payload-target status, DNA-damage-response pathway activity, and expression of drug-efflux transporters such as ABCB1 and ABCC1 [52,104].

Beyond identifying patients most likely to respond, biomarkers may also facilitate real-time monitoring of resistance evolution and help guide subsequent treatment decisions. Retrospective analyses of ADC sequencing have already demonstrated that shared targets and payload classes contribute to cross-resistance, supporting the rationale for mechanism-switching approaches that dual-payload ADCs seek to implement prospectively [28,43].

#### 2.6.5. Future Clinical Trial Designs

The ultimate test of dual-payload ADCs is whether mechanistic diversification translates into clinically meaningful improvements in durability of response. Achieving this objective may require clinical trial designs that differ from those traditionally used for ADC development.

Adaptive studies, biomarker-enriched cohorts, mechanism-switching crossover designs, and master protocols incorporating multiple resistance-defined populations may be particularly well suited to evaluating dual-payload strategies [33,40]. Such approaches can directly test whether the biological rationale underlying dual-payload ADCs translates into measurable clinical benefit and can help identify the patient populations in which that benefit is greatest.

More broadly, these trial designs reflect a broader shift in ADC development from target-driven therapy toward resistance-informed therapy. The central question is no longer simply whether an ADC can produce tumor responses, but whether it can produce more durable responses by addressing the biological mechanisms that limit existing treatments. The answer to that question will help define the clinical role of dual-payload ADCs and the validity of the broader translational framework described throughout this review.

## 3. PART III—General Lessons for Therapeutic Innovation

### 3.1. Beyond Dual-Payload ADCs: Generalizing the Translational Therapeutic Development Roadmap

The central premise of this review is that successful therapeutic innovation is often driven not by molecular format alone, but by the systematic translation of unmet clinical needs into mechanistically informed therapeutic solutions. Although dual-payload ADCs serve as the primary case study, the same developmental logic can be observed across many emerging therapeutic modalities. In each case, therapeutic advances have been driven by the identification of specific biological limitations, followed by the design of increasingly sophisticated interventions intended to address those limitations.

The examples discussed below illustrate how diverse therapeutic technologies often converge on remarkably similar development principles when confronted with analogous biological challenges. In this sense, the Translational Therapeutic Development Roadmap is not specific to ADCs, but reflects a broader framework for therapeutic innovation [13,33].

Although the seven-step roadmap is intended to provide a common conceptual framework across therapeutic modalities, the technical implementation of individual steps necessarily differs according to product class. In particular, validation and developability (Steps 5 and 6) are modality-specific processes. For example, ADCs emphasize conjugation chemistry, linker stability, and analytical characterization, whereas cell and gene therapies focus on cell fitness, vector manufacturing, potency assays, long-term persistence, and manufacturing logistics. Thus, the roadmap should be interpreted as defining a common translational logic rather than a uniform development workflow.

#### 3.1.1. Radiopharmaceutical Conjugates

Radiopharmaceutical therapies provide a clear example of resistance-informed therapeutic development. Agents such as ^177^Lu-PSMA-617 in metastatic prostate cancer and ^177^Lu-DOTATATE in neuroendocrine tumors were developed in response to the need for selective delivery of cytotoxic radiation to disseminated disease while minimizing systemic toxicity [119,120].

Importantly, these agents exploit a biological mechanism distinct from conventional ADCs. Whereas ADC efficacy depends on intracellular payload delivery, radionuclides generate crossfire radiation capable of killing neighboring antigen-low or antigen-negative cells independent of internalization [85]. This property directly addresses one of the key limitations associated with heterogeneous target expression.

The continuing exploration of radionuclides as ADC payloads further illustrates the convergence of therapeutic modalities and demonstrates how resistance-informed design can expand beyond conventional small-molecule warheads [85,121].

#### 3.1.2. Expanding Beyond Tumor Cell-Intrinsic Targeting: Tumor Microenvironment and Stromal-Targeting Therapeutics

Conventional antibody–drug conjugates (ADCs) primarily target antigens expressed on tumor cells and depend on efficient target binding, internalization, and intracellular payload release. However, an alternative therapeutic strategy is emerging that seeks to overcome biological barriers by targeting components of the tumor microenvironment rather than tumor cells themselves. Such approaches recognize that therapeutic resistance may arise not only from tumor-cell intrinsic mechanisms but also from the structural, spatial, and physiological organization of the tumor microenvironment [122,123,124,125,126].

One example is cancer stromal-targeting (CAST), which exploits the relative abundance and accessibility of extracellular matrix components across many solid tumors rather than relying exclusively on tumor-cell surface antigen expression [124]. Stromal-targeting ADCs therefore partially decouple therapeutic delivery from tumor-cell antigen heterogeneity, antigen loss, and inefficient internalization.

PYX-201, an ADC directed against the extra-domain B (EDB) splice variant of fibronectin—an oncofetal extracellular matrix protein largely absent from normal adult tissues—illustrates this strategy and has recently received FDA Fast Track designation. Preclinical studies have demonstrated that anti-EDB–fibronectin ADCs produce potent antitumor activity and may further enhance therapeutic efficacy when combined with immune checkpoint blockade [123]. Because EDB fibronectin is deposited throughout the tumor stroma rather than being restricted to individual tumor cells, such agents may overcome forms of spatial heterogeneity that are difficult for conventional tumor-cell-directed ADCs to address.

Unlike conventional internalizing ADCs, some stromal-targeting ADCs may function through localized proteolytic cleavage and extracellular payload release within the subendothelial matrix, generating a diffusible cytotoxic gradient capable of reaching neighboring antigen-negative tumor cells [125,126]. By engaging stromal structures that are often more uniformly distributed than tumor-associated antigens, these approaches may improve intratumoral drug distribution while reducing the impact of heterogeneous or low tumor-cell antigen expression.

Importantly, stromal-targeting ADCs are likely to complement rather than replace conventional tumor-cell-directed or dual-payload strategies. Whereas dual-payload ADCs seek to overcome resistance through the mechanistic diversification of cytotoxic payloads, stromal-targeting approaches address spatial and microenvironmental barriers that limit effective drug delivery. Together, these complementary strategies illustrate that resistance-informed therapeutic innovation may be achieved not only through new payloads and molecular architectures, but also through alternative biological targets and therapeutic delivery strategies.

More broadly, this evolution reinforces a central principle of the Translational Therapeutic Development Roadmap proposed in this review: successful therapeutic innovation begins with understanding the biological mechanisms that limit existing therapies and then selecting the molecular architecture, therapeutic modality, and biological target most appropriate for overcoming those limitations. In this framework, the choice of biological target becomes as important as the choice of payload, linker chemistry, or therapeutic platform itself.

#### 3.1.3. Multispecific Antibodies

Multispecific antibodies emerged in response to the recognition that single-target therapies often fail because tumors utilize multiple signaling pathways or evade treatment through antigen loss and pathway redundancy [127].

By simultaneously engaging two or more targets, multispecific antibodies attempt to address multiple biological vulnerabilities within a single therapeutic molecule. Applications include T-cell redirection, dual-pathway inhibition, co-targeting of escape antigens, and immune-cell recruitment.

The dual-target/dual-payload ADCs discussed in earlier sections represent a convergence between multispecific targeting and payload diversification. Similarly, emerging heterobifunctional antibody platforms are beginning to extend these concepts into targeted protein degradation and other novel therapeutic modalities [98,128].

Viewed through the roadmap framework, multispecific antibodies represent another example of therapeutic complexity arising not from technological ambition alone, but from the need to address biological complexity.

#### 3.1.4. Targeted Protein Degraders

Targeted protein degraders, including PROTACs and molecular glues, emerged from an important therapeutic limitation: many disease-driving proteins remain difficult or impossible to inhibit using conventional pharmacological approaches [84].

Rather than blocking protein function, degraders eliminate the target protein entirely through recruitment of endogenous degradation machinery. This approach expands the range of potentially druggable targets while introducing catalytic, sub-stoichiometric mechanisms of action.

The integration of degraders with targeted delivery systems has generated a rapidly growing class of degrader–antibody conjugates. Examples include anti-CD33 conjugates carrying GSPT1 degraders and ROR1-directed antibody–PROTAC conjugates capable of inducing BRD4 degradation in tumor cells [82,83,129,130].

These programs illustrate the same developmental sequence observed throughout this review: an unmet need is identified, a biological mechanism is understood, a mechanistic hypothesis is generated, and a therapeutic platform is engineered to address the underlying limitation.

#### 3.1.5. Cell and Gene Therapies

Cell and gene therapies provide perhaps the most striking examples of resistance-driven therapeutic evolution. Chimeric antigen receptor (CAR)-T-cell therapies have produced durable remissions in hematologic malignancies, yet their success has revealed new challenges including antigen loss, T-cell exhaustion, limited persistence, and immunosuppressive tumor microenvironments [131].

In response, next-generation CAR-T strategies increasingly incorporate multiple antigen specificities, engineered cytokine production, resistance to immune suppression, and additional functional modules designed to address specific mechanisms of treatment failure.

Gene therapies similarly originate from clearly defined biological hypotheses concerning disease-causing molecular defects and rely upon careful alignment between clinical need, mechanistic understanding, delivery technology, and long-term therapeutic objectives [132].

The parallels with dual-payload ADC development are notable. In both cases, increasingly sophisticated therapeutic architectures can be reviewed as responses to resistance and biological complexity.

#### 3.1.6. Future Multi-Mechanistic Therapeutics

**Figure 8:**
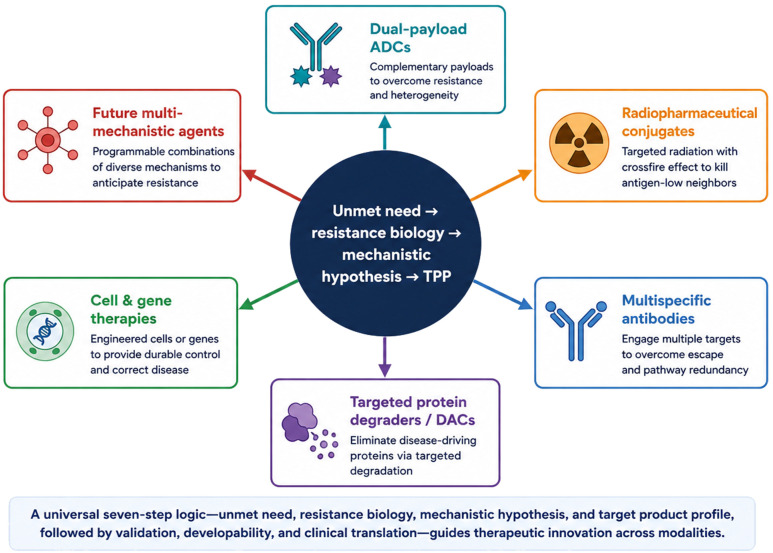
**The universal translational therapeutic development roadmap.** The same seven-step logic—unmet needs, resistance biology, mechanistic hypothesis, and target product profile, followed by validation, developability, and clinical translation—generalizes across dual-payload ADCs, radiopharmaceutical conjugates, multispecific antibodies, targeted protein degraders and degrader–antibody conjugates, cell and gene therapies, and future multi-mechanistic agents.

One emerging trajectory across modalities is from single-mechanism agents toward programmable, multi-mechanistic therapeutics that anticipate resistance by design—conjugates carrying complementary warheads, multispecifics engaging several targets, and combination modalities that integrate cytotoxic, degradative, radiative, and immune mechanisms [33,116] (Figure 8). Dual-payload ADCs are an early, instructive instance of this broader shift.

Importantly, the roadmap also disciplines this expansion against over-engineering. Each added mechanism must earn its place by solving a specific, documented failure mode and must survive the same developability and safety scrutiny as any other product attribute; complexity that does not map to a biological problem adds risk without benefit [31,33]. The convergence of conjugate chemistry, multispecific antibody engineering, targeted degradation, and radioligand therapy thus points toward a future of deliberately composed, resistance-aware therapeutics in which the modality is chosen to fit the biology rather than the reverse [13,116].

### 3.2. Conclusions

Therapeutic innovation is most likely to succeed when it follows a disciplined translational progression from an unmet clinical need to a developable therapeutic candidate. Throughout this review, dual-payload ADCs have served as a contemporary worked example of this process. Their emergence was driven by the growing post-ADC treatment gap, informed by increasingly detailed understanding of resistance biology, enabled by advances in conjugation and payload technologies, and supported by preclinical studies and early-stage clinical programs designed to address specific mechanisms of therapeutic failure.

Importantly, the significance of dual-payload ADCs extends beyond the modality itself. They illustrate how biological insight, molecular engineering, developability considerations, and clinical strategy can be integrated into a coherent translational therapeutic development pathway. Whether applied to ADCs, radiopharmaceuticals, multispecific antibodies, targeted protein degraders, cell therapies, or future therapeutic platforms, the same fundamental principle remains: successful medicines are most often those designed not only to attack disease, but also to anticipate and overcome the mechanisms through which disease adapts and survives.

Viewed through this lens, dual-payload ADCs should be regarded not simply as an emerging molecular format, but as an example of resistance-informed therapeutic innovation. Although current evidence remains largely preclinical and early clinical, they illustrate how resistance biology can be translated into mechanistic hypothesis, molecular architecture, pharmaceutical development, and ultimately clinical strategy. Their future clinical value will be determined by ongoing and future clinical studies designed to establish whether these conceptual advantages translate into meaningful improvements in patient outcomes [93,96].

More broadly, the principal conclusion of this review is that therapeutic innovation should not begin with a molecule—it should begin with a clearly defined unmet clinical need. Resistance biology should be viewed not merely as a consequence of therapeutic failure, but as a primary design input for next-generation therapeutics. The resistance-informed Translational Therapeutic Development Roadmap proposed here provides an organizing framework for systematically integrating unmet clinical needs, disease biology, mechanistic hypothesis, molecular design, developability, manufacturability, and clinical translation. Dual-payload ADCs represent a particularly instructive example of this approach because their molecular architecture directly reflects biological hypotheses regarding therapeutic resistance. Ultimately, the success of future therapeutics will depend not on increasing molecular complexity alone, but on selecting the right biological problem, designing the appropriate therapeutic solution, and maintaining translational alignment throughout the entire drug development process.

### 3.3. Key Messages

The key messages of this study are as follows:

1. Therapeutic innovation should begin with a clearly defined unmet clinical need, not with a molecule or a molecular target.
2. Resistance biology should be viewed not merely as a cause of therapeutic failure, but as a primary design input for next-generation therapeutic innovation.
3. Successful therapeutic development requires continuous alignment among unmet clinical needs, disease biology, mechanistic hypothesis, Target Product Profile (TPP), molecular design, developability, and clinical translation.
4. Molecular complexity is justified only when it addresses a clearly defined biological problem. Developability and manufacturability should therefore be considered integral design constraints rather than downstream development activities.
5. Dual-payload ADCs illustrate the broader Translational Therapeutic Development Roadmap, demonstrating how resistance-informed design can be systematically translated into next-generation therapeutics across multiple therapeutic modalities.

## Figures and Tables

**Table 1:** **Illustrative target product profile (TPP) for a next-generation dual-payload ADC.** Each attribute translates a resistance-biology insight into a measurable product requirement that downstream design, validation, and manufacturing must satisfy.

TPP Attribute	Target Requirement	Biological/Clinical Rationale
Indication/line	Solid tumors progressing after a TOP1-inhibitor ADC (post-Enhertu/post-Trodelvy)	Defines the unmet population with payload cross-resistance
Post-ADC activity	Objective responses in TOP1i-refractory and antigen-low models and patients	Direct test of mechanistic differentiation
Resistance coverage	Retained potency under P-gp/ABCC1 efflux and heterogeneous antigen expression	Counters dominant network nodes (efflux, heterogeneity)
Safety	Manageable myelosuppression; low ILD/pneumonitis signal; predictable off-target payload toxicity	Enables durable, repeat-dose therapy
Dosing	Q3W intravenous infusion, outpatient compatible	Aligns with established ADC practice and adherence
Manufacturability	Defined DAR and fixed payload ratio; >90% conjugation; stable, scalable process	Ensures a reproducible, regulatable product

**Table 2:** Resistance-guided payload pairing strategies for dual-payload ADCs, summarizing the biological rationale, resistance mechanisms addressed, potential advantages, and key development and formulation considerations. Combinations are illustrative and hypothesis-generating rather than clinically validated.

Payload Pairing Strategy	Biological Rationale	Resistance Mechanism(s) Addressed	Potential Advantages	Key Development/Formulation Challenges
Topoisomerase I inhibitor + tubulin inhibitor	Combines DNA-replication stress with mitotic-spindle disruption, engaging two independent cell-cycle–dependent death pathways.	Payload-class resistance to a single mechanism; heterogeneous proliferative states within the tumor.	Broad activity across cycling and slowly cycling cells; potential bystander coverage from a membrane-permeable topoisomerase I payload.	Overlapping myelosuppression; differing potencies require careful DAR and payload-ratio control; linker compatibility for two chemistries.
Topoisomerase I inhibitor + DNA-damage-response (DDR) inhibitor (e.g., ATR/PARP)	DDR inhibition prevents repair of topoisomerase I–induced lesions, converting sublethal into lethal DNA damage (synthetic-lethal logic).	Up-regulated DNA-repair capacity; adaptive tolerance to DNA-damaging payloads.	Mechanistic synergy at low payload exposure; may lower the effective cytotoxic dose and widen the therapeutic window.	Sequence- and timing-dependence of synergy; on-target DDR toxicity; need to demonstrate true synergy rather than additivity.
Cytotoxic payload + immunomodulatory payload	Pairs direct tumor-cell killing with local immune activation (e.g., immunogenic cell death, innate-immune agonism) to recruit host antitumor immunity.	Immune-evasive or immunologically ‘cold’ microenvironments; residual disease after cytotoxic pressure.	Potential for durable immune-mediated responses beyond direct cytotoxicity; possible coverage of antigen-low cells.	Divergent PK and exposure requirements of the two classes; immune-related toxicities; complex PD and biomarker readouts.
Cell-permeable + non-cell-permeable payload	Retains a non-permeable payload within the antigen-positive cell while a permeable payload diffuses to neighboring antigen-low cells (bystander effect).	Antigen heterogeneity and partial antigen loss; mixed payload sensitivity.	Simultaneous coverage of antigen-high and antigen-low compartments from a single construct.	Spatial decoupling may expose neighboring cells to a single agent (local selection pressure); requires validation in spatially heterogeneous models.

**Table 3:** **Representative Dual-Payload ADC Programs by Development Stage and Therapeutic Logic (2024–2026):** Programs are drawn from peer-reviewed reports, conference disclosures, and company communications; development status evolves rapidly and should be reverified against current registries before citation.

Program	Company (Country)	Target/Format	Payload Pair/Pairing	Resistance Axis Addressed	Stage
Clinical (Phase 1)					
KH815	Chengdu Kanghong (CN)	TROP2 · dual-payload	TOP1i + RNA Pol II inhibitor	Transcriptional escape; DNA-damage backup	Phase 1
KHN922	Chengdu Kanghong (CN)	HER3 · dual-payload	TOP1i + RNA Pol II inhibitor	Transcriptional escape; heterogeneity	Phase 1
IBI3020	Innovent Biologics (CN)	CEACAM5 · dual-payload	Two cytotoxic payloads (DuetTx)	Heterogeneity; payload-specific resistance	Phase 1
IBI3028	Innovent Biologics (CN)	EGFR/c-Met · bispecific dual-payload	Two payloads (n.d.)	Antigen heterogeneity (dual antigen) + payload	Phase 1
CLIO-8221	Callio Therapeutics (US)	HER2 · dual-payload	TOP1i (exatecan) + ATR inhibitor (berzosertib)	TOP1 mutation; DNA-repair adaptation	Phase 1
SHR-A2102 *	Hengrui Pharma (CN)	Nectin-4 · ADC	TOP1i (single-payload per public data)	(listed in source; confirm dual-payload)	Phase 1
IND filed/accepted/cleared					
CAN016	CanWell Pharma (US/CN)	HER2 · dual-payload	Two cytotoxic MoAs (StarLinker platform)	Heterogeneity; post-ADC resistance	IND cleared (FDA)
DXC018	Hangzhou DAC Biotech (CN)	HER2 ECD2 + ECD4 · biparatopic dual-payload	TOP1i + antimetabolite	TOP1 mutation; antigen heterogeneity	IND accepted
JSKN021	Alphamab Oncology (CN)	EGFR/HER3 · 2-in-1 bispecific dual-payload	TOP1i (T01) + MMAE (tubulin)	TOP1 mutation; heterogeneity	IND accepted
Preclinical/platform—China					
DB-1419	DualityBio (CN)	EGFR + B7-H3 · bispecific dual-payload	Two MOAs (DIBAC; novel toxin class)	Heterogeneity; immune/TME escape	Preclinical
GQ1009	GeneQuantum (CN)	HER2 · dual-payload	TOP1i + tubulin inhibitor	TOP1 mutation; efflux	Preclinical
SKB571	Kelun-Biotech (CN)	Bispecific dual-payload (targets n.d.)	Two synergistic toxins	Heterogeneity; payload resistance	Preclinical
(platform)	Affinity Biosciences (CN)	Dual-payload	TOP1i + TLR7/8 agonist	Immune exclusion; TME escape	Preclinical
(platform)	ARisGen (CN)	Dual-payload	Non-TOP1/non-tubulin toxins	Cross-resistance avoidance	Preclinical
(undisclosed)	Hengnuokang (CN)	n.d.	n.d.	n.d.	Preclinical
CD276-ADC	Academic (Zhou et al.)	CD276/B7-H3 · dual-payload	Cytotoxic + immune activator	Immune exclusion	Preclinical
Preclinical/platform—global (ex-China)					
SPX-629	SparX Biopharmaceutical	HER2 · dual-payload	TOP1i + tubulin inhibitor	TOP1 mutation; ABCG2 efflux	Preclinical → IND
SPX-602	SparX Biopharmaceutical	VEGF/PD-L1 · dual-payload	TOP1i + tubulin inhibitor	TOP1 mutation; efflux; TME/angiogenesis	Preclinical → IND
SPX-608	SparX Biopharmaceutical	Immune-cell-targeting · dual-payload	TOP1i + tubulin inhibitor	TOP1 mutation; efflux; immune engagement	Preclinical → IND
(multiple)	Sutro Biopharma (US)	Site-specific high-DAR dual-payload	Various high-DAR pairs	Efflux; heterogeneity	Preclinical
(multiple)	Araris Biotech (CH)	HER2, NaPi2b · dual/multi-payload	Two TOP1 inhibitors	TOP1 dose-intensification; heterogeneity	Preclinical
(platform)	Avacta (UK)	TME-activated dual-payload	Two payloads (CISION)	TME escape; selective release	Preclinical
(research)	Pfizer/Seagen (US)	Research · dual-payload	MMAE + MMAF (permeable + non-permeable)	Heterogeneity; bystander coverage	Foundational
(academic)	Tsuchikama lab, UTHealth (US)	HER2 · dual-payload	MMAE + MMAF; also ^177^Lu + antimitotic	Heterogeneity; drug resistance	Foundational

## Data Availability

No new data were generated or analyzed in this study. Data sharing is not applicable to this article, as it is based exclusively on the review and interpretation of the previously published literature.

## Abbreviations

ABC: ATP-binding cassette; ADC, antibody–drug conjugate; CAR, chimeric antigen receptor; DAR, drug-to-antibody ratio; DAC, degrader–antibody conjugate; DDR, DNA-damage response; DBCO, dibenzocyclooctyne; HER2, human epidermal growth factor receptor 2; iADC, immunostimulatory ADC; ILD, interstitial lung disease; MMAE/MMAF, monomethyl auristatin E/F; ncAA, non-canonical amino acid; PARP, poly(ADP-ribose) polymerase; PK/PD, pharmacokinetics/pharmacodynamics; PROTAC, proteolysis-targeting chimera; QTPP, quality target product profile; STING, stimulator of interferon genes; TCO, trans-cyclooctene; TLR, Toll-like receptor; TOP1, topoisomerase I; TPP, target product profile; TROP2, trophoblast cell-surface antigen 2.
